# Structural and dynamical strategies to prevent runaway excitation in reservoir computing

**DOI:** 10.3389/fncom.2026.1845838

**Published:** 2026-08-14

**Authors:** Claus Metzner, Achim Schilling, Andreas Maier, Thomas Kinfe, Patrick Krauss

**Affiliations:** 1 Cognitive Computational Neuroscience Group, Pattern Recognition Lab, Friedrich-Alexander-University Erlangen-Nürnberg (FAU), Erlangen, Germany; 2 Neuromodulation and Neuroprosthetics, University Hospital Mannheim, University Heidelberg, Mannheim, Germany; 3 Neuroscience Lab, University Hospital Erlangen, Erlangen, Germany; 4 BG Klinik Ludwigshafen, Ludwigshafen am Rhein, Germany

**Keywords:** dynamical stability, dynamical systems, edge of chaos, homeostatic regulation, recurrent neural networks, reservoir computing

## Abstract

Reservoirs, typically implemented as recurrent neural networks (RNNs) with fixed random connection weights, can be combined with a simple trained readout layer to perform a wide range of computational tasks. However, increasing the magnitude of reservoir connection weights to exploit non-linear dynamics can cause the network to develop strong spontaneous activity that drives neurons into saturation, dramatically degrading performance. In this work, we investigate two distinct countermeasures against such runaway excitation. The first approach introduces a subtle non-homogeneous structure into the matrix of connection weights. *w*_*ij*_, without altering the overall probability distribution *p*(*w*). We identify several favorable structuring principles, such as creating a small subset of neurons with weaker-than-average input connections. Even if the rest of the reservoir falls into runaway saturating behavior, this weakly coupled subset remains in a mildly non-linear regime whose dynamics can still be exploited by the readout layer. The second approach implements a form of automatic gain control (AGC), in which a dedicated control unit dynamically regulates the reservoir's average global activation toward an optimal setpoint. Although the control unit modulates the excitability of the reservoir only via a global gain factor, this mechanism substantially enlarges the dynamical regime favorable for computation and renders performance largely independent of the underlying connection statistics.

## 1 Introduction

Deep learning has made substantial progress in recent years (LeCun et al., 2015; Alzubaidi et al., 2021), partly propelled by the emergence of large language models (Min et al., 2023). These models primarily depend on feedforward architectures, where information flows unidirectionally from input to output. Recurrent neural networks (RNNs), in contrast, feature feedback connections that enable them to function as self-sustaining dynamical systems (Maheswaranathan et al., 2019), maintaining internal activity without requiring continuous external input.

Several universal capabilities characterize RNNs, including their theoretical capacity to approximate arbitrary mappings (Schäfer and Zimmermann, 2006) and replicate general dynamical systems (Aguiar et al., 2023). These properties, along with their practical advantages, have generated sustained interest in understanding the internal mechanisms of these networks. RNNs can integrate information over prolonged temporal intervals (Jaeger, 2001; Schuecker et al., 2018; Büsing et al., 2010; Dambre et al., 2012; Wallace et al., 2013; Gonon and Ortega, 2021), and they possess the ability to construct compact yet rich internal representations through a balance of dimensional compression and expansion (Farrell et al., 2022).

Research has also examined how RNN dynamics can be controlled and stabilized, especially when subjected to noise from both internal and external sources (Rajan et al., 2010; Jaeger, 2014; Haviv et al., 2019; Molgedey et al., 1992; Ikemoto et al., 2018; Krauss et al., 2019a; Bönsel et al., 2021; Metzner and Krauss, 2022). RNNs have additionally attracted interest as computational frameworks for brain-like information processing (Barak, 2017). Sparse RNNs with limited connections per unit, which mirror the structural organization of biological neural circuits (Song et al., 2005), have demonstrated benefits for memory retention and representational efficiency (Brunel, 2016; Narang et al., 2017; Gerum et al., 2020; Folli et al., 2018).

Trainable recurrent architectures commonly regulate information flow by learned, unit-specific gates. Long short-term memory networks introduced explicit input, output, and forget gates (Hochreiter and Schmidhuber, 1997), and more recent hierarchical and extended LSTM variants refine this principle for long-sequence modeling (Qin et al., 2023; Beck et al., 2024). The global activity feedback considered here serves a different purpose. It is a single scalar controller applied online to an otherwise fixed random reservoir; it neither learns neuron-specific gates nor replaces a task-trained recurrent architecture. Our comparison is therefore conceptual rather than competitive: both approaches regulate recurrent information flow, but at very different spatial resolution and with different training requirements.

Our earlier work established a systematic investigation into how structural features influence RNN dynamics, beginning with small three-neuron motifs (Krauss et al., 2019c). Extending this foundation, we showed how large autonomous networks can be controlled through three critical parameters: weight distribution width *w*, connection density *d*, and excitation/inhibition balance *b* (Krauss et al., 2019b; Metzner and Krauss, 2022). We further explored how these systems respond to noise, uncovering resonance-like phenomena and stochastic stabilization effects (Bönsel et al., 2021; Schilling et al., 2022; Krauss et al., 2016, 2019a; Schilling et al., 2021, 2023; Metzner et al., 2024).

Our more recent research has investigated how particular dynamical characteristics and structural regularities affect reservoir computing system performance.

In Metzner et al. (2025a), we systematically manipulated the degree of neuronal non-linearity and the intensity of recurrent coupling to examine their effects on classification accuracy in synthetic tasks. Remarkably, even “quiet” reservoirs exhibiting minimal internal dynamics and extremely weak non-linearity demonstrated the capacity to produce linearly separable representations for the readout layer, provided that subtle high-dimensional state features were leveraged. Although task performance generally declined under highly chaotic dynamics, accuracy frequently peaked near transition regions between chaotic and oscillatory or fixed-point regimes, providing support for the “edge of chaos” hypothesis.

In Metzner et al. (2025b), we examined how biologically motivated structural regularities influence both the autonomous dynamics of RNNs and their functional performance in reservoir computing. These regularities included Dale's principle, the creation of stronger and weaker patches in the connection weight matrix to create local heterogeneity (Sporns and Betzel, 2016; Meunier et al., 2010), as well as reciprocal symmetry similar to Hopfield networks (Hopfield, 1982). Our findings indicated that Dale-conforming output weights and heterogeneous connectivity patches tend to improve task accuracy, whereas strong reciprocal symmetry proved detrimental, frequently pushing the system toward premature saturation and diminishing its ability to discriminate between inputs. Collectively, these results already hint at potential advantages of incorporating organizational structure into randomly connected networks.

These questions are related to three broader themes. First, fading memory acts as an inductive bias in recurrent systems (Dubinin and Effenberger, 2024): useful computation requires past inputs to persist, but arbitrary initial conditions and obsolete inputs must eventually be forgotten. Second, heterogeneity can broaden parameter regions with critical-like behavior rather than merely shifting a single critical point (Sánchez-Puig et al., 2023). Third, reservoir design is constrained by a task-dependent trade-off between memory and non-linear transformation, although suitable architectures can partly move beyond a simple one-dimensional compromise (Inubushi and Yoshimura, 2017). In the present setting, weakly coupled reservoirs favor stable input tracking, whereas increasing recurrent drive adds memory and non-linearity but also increases the risk of chaos and tanh saturation.

In the present work, we focus on the problem of runaway excitations in strongly connected reservoir networks, which can arise independently of the present input. These excitations drive neurons deep into the saturation of their sigmoidal activation functions and manifest at the network level as global synchronous oscillations, chaotic fluctuations, or global fixed points. In any of these cases, the computational performance of the reservoir computer (RC) typically degrades dramatically because the dynamics is no longer predominantly determined by the present input. To address this problem, we test two different approaches.

Our first approach is to start with a statistically homogeneous connection weight matrix and then to re-distribute its elements in order to create heterogeneous structure in the matrix, yet without altering the global probability density distribution of the elements. The idea is that runaway excitations can often be interpreted as a herding effect driven by strong negative or positive feedback between all neurons in the reservoir. By creating diverse subsets of neurons with different statistical properties, such global herding effects may be suppressed.

Our second approach is a direct dynamical regulation of network activity. We compute a running average of the overall network activation level, measured as the root-mean-squared activation of all reservoir neurons. A control unit then regulates a global prefactor of the weight matrix to keep the activity close to a setpoint that has been optimized for computation. Although highly simplified, this mechanism bears a conceptual resemblance to global regulatory processes in biological neural systems, where overall excitability and effective coupling strength are modulated by neuromodulators, hormones, or glial interactions. While our model does not attempt to capture such mechanisms in detail, it illustrates how a coarse-grained, global control signal can stabilize network dynamics and expand the regime of useful computation.

Feedback regulation has also been combined with residual recurrent connections to improve the practical training of equilibrium-propagation networks (Chen and Liu, 2026), building on the relation between contrastive Hebbian learning and backpropagation (Xie and Seung, 2003). Our objective is distinct: we do not use feedback to compute a learning signal or modify the recurrent weights. Instead, the controller homeostatically rescales a fixed random recurrent matrix during inference.

## 2 Methods

### 2.1 Generation of a homogeneous weight matrix

The weight matrix **W** of the reservoir's recurrent connections is random but controlled by three statistical parameters: the density *d* of non-zero connections, the excitatory/inhibitory balance *b*, and the recurrent coupling strength *w*, which is defined by the standard deviation of the Gaussian distribution of weight magnitudes.

The density *d* ranges from *d* = 0 (isolated neurons) to *d* = 1 (fully connected network). The balance *b* ranges from *b* = −1 (purely inhibitory connections) to *b* = +1 (purely excitatory connections), with *b* = 0 corresponding to a perfectly balanced system. The coupling strength *w* can take any positive value, where *w* = 0 corresponds to unconnected reservoir neurons.

The bias terms ***b*_*w*_** applied to the reservoir neurons are also drawn from a Gaussian distribution with zero mean and a fixed variance, ensuring that the reservoir operates in a dynamic regime suitable for information processing.

In order to construct an *N*×*N* weight matrix with given parameters (*b, d, w*), we proceed as follows:

First, we generate a matrix **M**^(*magn*)^ of weight magnitudes by drawing the *N*^2^ matrix elements independently from a zero-mean normal distribution with standard deviation *w* and then taking the absolute value.

Next, we generate a random binary matrix **B**^(*nonz*)^ ∈ {0, 1}^*N*×*N*^, where the probability of a matrix element being 1 is given by the density parameter *d*, that is, *p*_1_ = *d*.

We then generate another random binary matrix **B**^(*sign*)^ ∈ {−1, +1}^*N*×*N*^, in which the probability of a matrix element being +1 is given by


$$\begin{array}{l} p_{+1}=\frac{1+b}{2} \end{array}$$ (1)

where *b* is the balance parameter. The relation between the balance parameter and this probability is given in Equation 1.

Finally, the weight matrix is constructed by element-wise multiplication:


$$\begin{array}{l} W_{mn}=M_{mn}^{\left(magn\right)}\cdot B_{mn}^{\left(nonz\right)}\cdot B_{mn}^{\left(sign\right)} \end{array}$$ (2)

The resulting weight matrix is defined in Equation 2. Unless explicitly stated otherwise, the density parameter is set to its maximum value *d* = 1. The sparse-connectivity control experiment in Supplemental material additionally considers *d* = 0.1, 0.2, and 0.5.

The biases *b*_*w, n*_ of the reservoir are drawn independently from a zero-mean normal distribution with a standard deviation of *w*′. Note that throughout this paper, the strength of the reservoir bias is always set to *w*′ = 0.1. Thus, even for an uncoupled reservoir with *w* = 0 and zero input, the neurons will have non-zero resting values due to the random biases.

### 2.2 Generation of a structured weight matrix

Starting from a statistically homogeneous weight matrix **W** of size *N*×*N*, generated under fixed parameters width *w*, density *d*, and balance *b*, our goal is to introduce controlled heterogeneity and structural organization without altering the overall distribution of weight values. To this end, we apply a three-step procedure consisting of mask generation, element sorting, and structured reassembly.

In the first step, a binary mask of the same shape as **W** is constructed. The mask entries take values 0 or 1, and their spatial arrangement is determined by a mode parameter mskMod. In *random* mode, a fraction oneFrc of all matrix elements is selected uniformly at random and marked with 1. In *rows* mode, a fraction oneFrc of all rows is randomly selected and entirely marked with 1. In *cols* mode, a fraction oneFrc of all columns is randomly selected and entirely marked with 1. In *diagBlocks* mode, the mask consists of a chain of contiguous *S*×*S* blocks of ones arranged along the main diagonal; this requires **W** to be square and its dimension to be divisible by *S*. In this latter case, the parameter oneFrc is not used.

In the second step, all elements of **W** are collected into a one-dimensional list and sorted according to a mode parameter srtMode. Sorting can be performed with respect to the raw weight values or their absolute magnitudes, each in either ascending or descending order. This step establishes a controlled ranking of weights while preserving the original multiset of values and hence the distribution implied by *w*, *d*, and *b*.

In the final step, the sorted list of weight values is redistributed over the matrix positions according to the previously generated mask. The number of elements assigned to mask entries equal to 1 and 0 is determined by their respective counts in the mask. The sorted list is split accordingly into two subsets, which are randomly permuted within themselves to avoid introducing unintended fine-scale order. The shuffled subsets are then placed into the matrix positions corresponding to mask entries 1 and 0, respectively.

The overall procedure thus preserves the global weight distribution exactly, while introducing structured heterogeneity at the level specified by mskMod, oneFrc, *S*, and srtMode. This allows us to generate systematically structured weight matrices from an initially homogeneous ensemble without modifying the underlying statistics of the weights.

### 2.3 Design of reservoir computer (RC)

The RC consists of an input layer, a recurrent reservoir, a readout layer and optionally, a control unit. The input data comprises *E* consecutive episodes, each representing, for example, a pattern to be classified, or a priming sequence triggering the output of a specific target response. This data stream is fed into the reservoir and circulates through the system while propagating toward the output.

At each time step *t*, the input layer receives *M* parallel signals $x_{m}^{\left(t\right)}\in \left[-1,+1\right]$. These are linearly transformed by the input matrix **I** of size *N*×*M* and injected into the reservoir, as described by Equation 3. Each input episode spans *T* time steps.

The input layer consists solely of the matrix **I** and is therefore purely linear. Its elements *I*_*mn*_ are drawn independently from a normal distribution with zero mean and standard deviation *w*_*I*_. To study autonomous RNN dynamics, we set *w*_*I*_ = 0, effectively decoupling the reservoir from external input.

The reservoir comprises *N* recurrently connected neurons with tanh activation. At each time step, all neuron states *y*_*n*_ are updated in parallel. Each neuron receives a bias term *b*_*w, n*_, input from the external signals via **I**, and recurrent input via the weight matrix **W** (see Equation 3), which can be dynamically modulated by a gain factor *g* from the control unit. In settings without control, we set *g* = 1. Initial states $y_{n}^{\left(0\right)}$ are drawn uniformly from [−1, +1] and kept fixed for repeated simulations of the same reservoir. Different weight matrices receive independent initial states.

The readout layer performs an affine-linear transformation of the reservoir states *y*_*n*_ using a *K*×*N* output matrix **O** and a bias vector ***b***_*o*_, as in Equation 4. These parameters are trained via the pseudoinverse method (see below).

In the sequence generation task, which serves as the main information processing benchmark in this work, the continuous outputs *z*_*k*_ of the readout layer directly form the vectors of the output sequence. In classification tasks, by contrast, the *z*_*k*_ represent soft votes that are converted into discrete predicted class labels *c* using the argmax function. This final step introduces a non-linearity that sharpens the class boundaries in the output space.

In summary, the RC and the conversion of its continuous outputs into class labels are governed by Equations 3–5.


$$\begin{array}{l} y_{n}^{\left(t\right)}=\tanh\left(b_{w,n}+\underset{m}{\sum }I_{nm}x_{m}^{\left(t-1\right)}+g^{\left(t\right)}\underset{n^{\prime }}{\sum }W_{nn^{\prime }}y_{n^{\prime }}^{\left(t-1\right)}\right) \end{array}$$ (3)

$$\begin{array}{l} z_{k}^{\left(t\right)}=b_{o,k}+\underset{n}{\sum }O_{kn}y_{n}^{\left(t\right)} \end{array}$$ (4)

$$\begin{array}{l} c^{\left(t\right)}=arg max\left\{z_{k}^{\left(t\right)}\right\} \end{array}$$ (5)

### 2.4 Unit for automatic gain control (AGC)

The control unit computes a running average ${\bar{A}}^{\left(t\right)}$ of the reservoir's momentary root-mean-square activation *A*^(*t*)^. The momentary and running-average activities are defined in Equations 6 and 7, respectively.


$$\begin{array}{l} A^{\left(t\right)}=\sqrt{\frac{1}{N}\underset{n}{\sum }{\left(y_{n}^{\left(t\right)}\right)}^{2}} \end{array}$$ (6)

$$\begin{array}{l} {\bar{A}}^{\left(t\right)}=\mu A^{\left(t\right)}+\left(1-\mu \right){\bar{A}}^{\left(t-1\right)} \end{array}$$ (7)

The RMS is sign independent and invariant to a permutation of neuron labels, as required for a global population signal. Squaring also gives comparatively large activation excursions greater influence. For tanh neurons this is useful because large absolute activations indicate approach to the saturated regime in which small input-related differences are suppressed.

At time zero, we set ${\bar{A}}^{\left(0\right)}=0$. The mixing factor μ, ranging between 0 and 1, controls how strongly the present network activation is weighted compared to the past average. Equation 7 is an exponential low-pass filter: small μ prevents the controller from reacting strongly to rapid task-related fluctuations, whereas larger μ follows changes in population activity more quickly.

The running average ${\bar{A}}^{\left(t\right)}$ is compared to an activity setpoint α, which in our system has a range from 0 to 1. Depending on whether the difference is positive or negative, the current gain factor *g* is multiplied with an exponential function that is smaller or larger than one, respectively. The gain update is given in Equation 8.


$$\begin{array}{l} g^{\left(t\right)}=g^{\left(t-1\right)}\exp\left(-\epsilon \left({\bar{A}}^{\left(t\right)}-\alpha \right)\right) \end{array}$$ (8)

At time zero, we set *g*^(0)^ = 1. The positive feedback parameter ϵ determines the sensitivity of the gain control mechanism. The exponential multiplicative update guarantees *g*^(*t*)^>0 and changes the gain proportionally to its current value. If activity exceeds the setpoint, gain is reduced; if activity falls below it, gain is increased. The update therefore implements negative feedback around α. In the special case ϵ = 0, automatic gain control is switched off.

**Figure 1:**
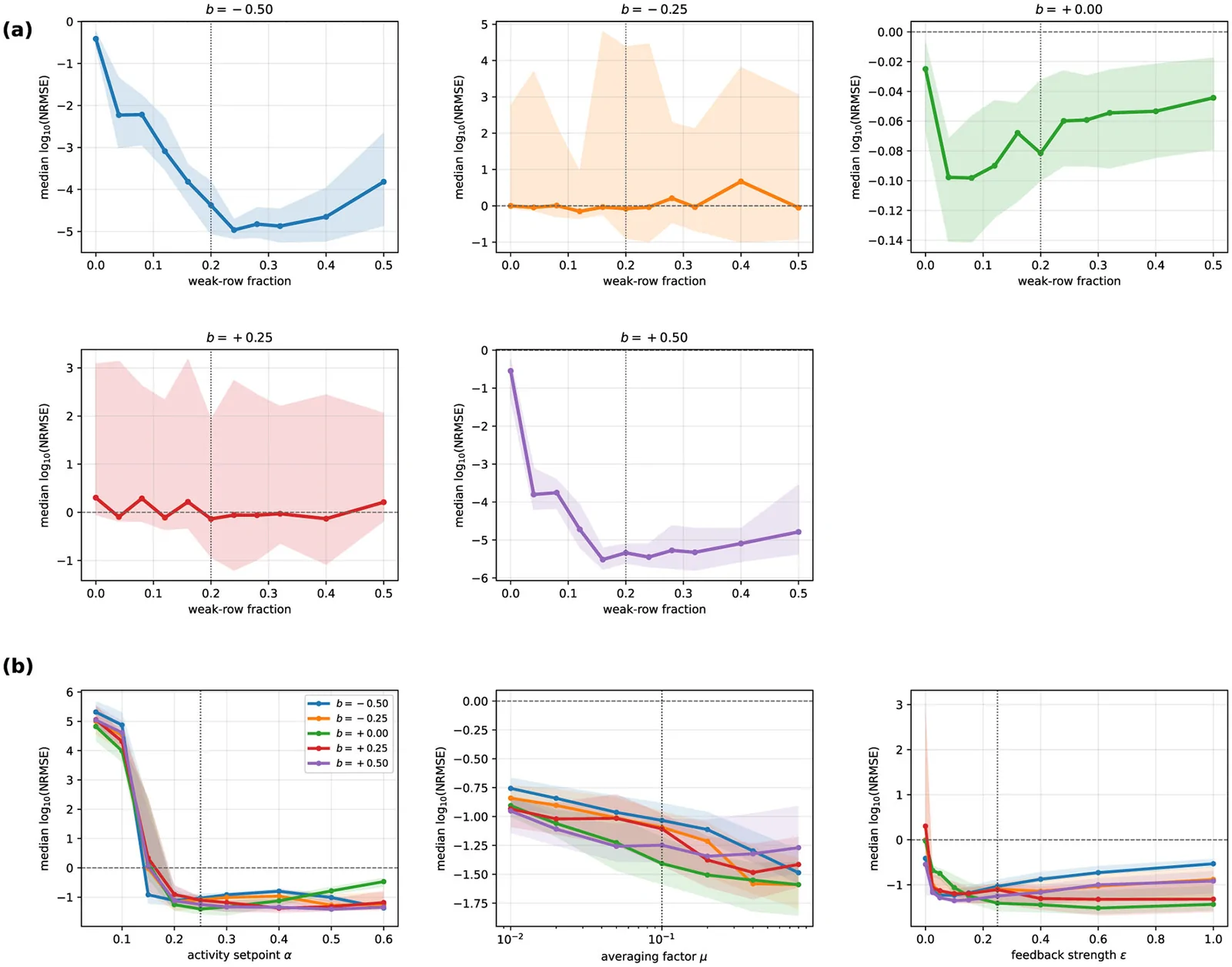
Parameter ablations for weak-row structuring and automatic gain control on the sequence-generation task. **(a)** Weak-row-fraction ablation at five representative excitatory/inhibitory balances, *b* = −0.50, −0.25, 0, +0.25, and +0.50. Each panel shows the median log_10_(NRMSE) as a function of the fraction of weak rows; shaded regions indicate the interquartile range over 30 paired random realizations. The horizontal dashed line marks NRMSE = 1, and the vertical dotted line marks the weak-row fraction 0.20 used in the main analyses. Separate vertical scales are used because typical errors differ by several orders of magnitude among balance regimes. **(b)** One-at-a-time ablations of the AGC activity setpoint α (left), averaging factor μ (center), and feedback strength ϵ (right). Colored curves correspond to the same five balance values as in **(a)**, and shaded regions indicate interquartile ranges over 30 paired realizations. In each scan, the other two AGC parameters are fixed at their standard values, α = 0.25, μ = 0.10, and ϵ = 0.25. Vertical dotted lines identify the respective standard values; the μ axis is logarithmic. The horizontal dashed line again marks NRMSE = 1.

The results with AGC shown in this paper were based on the following parameter settings: μ = 0.1, α = 0.25 and ϵ = 0.25. Here, α selects the desired activity scale, μ the temporal resolution of the activity estimate, and ϵ the correction rate. The one-at-a-time scans in Figure 1b show broad effective ranges around these standard values. They support their use as robust working settings for the present experiments, but do not establish a unique or task-independent optimum.

### 2.5 Sequence generation task

In this task, the reservoir computer functions as a deterministic system that maps an input sequence *X* of real-valued vectors onto a corresponding output sequence *Z*:


$$X\in {\left[-1,+1\right]}^{TI\times M}\to Z\in {\left[-1,+1\right]}^{TO\times K}$$

Here, *TI* and *TO* denote the temporal lengths of the input and output sequences, and *M* and *K* their respective vector dimensions.

At the beginning of each episode, a randomly chosen input sequence *X* from one of *N*_*DC*_ discrete classes is fed into the network, driving it into a class-specific internal configuration or “priming state.” After the input ends, the reservoir evolves autonomously through a sequence of internal states *Y*, which the linear readout layer is trained to map onto the corresponding target sequence *Z*.

Since the system is strictly deterministic, it will always produce the same trajectory for each distinct priming state. Provided that the induced trajectory is not a cyclic attractor with a period shorter than *TO*, the readout should in principle be able to convert the trajectory into the desired output sequence.

However, because the reservoir is not reset at the beginning of each episode, residues of previous states may persist, such that the priming state is not exactly identical every time a given class is selected. If the reservoir operates in a chaotic regime, these small differences can be amplified, producing an effectively unpredictable trajectory that cannot be mapped to the correct target.

In practice, the target mapping also fails if the reservoir neurons enter the saturated “digital” state, since the resulting trajectory is insufficiently rich and lacks the necessary high-dimensional diversity.

Successful performance therefore requires a balance between stability and richness: the reservoir must forget prior excitation rapidly enough to respond reproducibly to identical inputs, while still maintaining sufficient temporal and spatial diversity across neurons.

To systematically explore the influence of network parameters under controlled conditions, we employ a minimal task configuration with *M* = *K* = 2, *TI* = 1, *TO* = 2, and *N*_*DC*_ = 2. The class-specific input sequences *X*_*c*_ and their target sequences *Z*_*c*_ are predefined, with all vector components drawn independently from a uniform distribution over [−1, +1]. In each episode, one of the *N*_*DC*_ cases is selected at random.

This deliberately minimal benchmark is strongly driven by the current one-step priming input and has only a short output horizon. It is therefore sensitive to spontaneous or saturated dynamics that overwrite input-related differences, but it does not by itself establish long-term memory capacity or broad task generality. NARMA-10 is included as a complementary test with a longer non-linear temporal dependency.

### 2.6 NARMA-10 task

As a complementary benchmark requiring both non-linear processing and temporal memory, we use the tenth-order non-linear autoregressive moving-average (NARMA-10) task. It consists of a scalar input *u*_*t*_ drawn independently from a uniform distribution over [0, 0.5] and a scalar target *s*_*t*_. Starting from *s*_*t*_ = 0, the target is generated by the conventional recurrence. This recurrence is given in Equation 9.


$$\begin{array}{l} s_{t+1}=0.3s_{t}+0.05s_{t}\overset{9}{\underset{j=0}{\sum }}s_{t-j}+1.5u_{t-9}u_{t}+0.1. \end{array}$$ (9)

Unlike the episodic sequence-generation task, NARMA-10 is treated as one uninterrupted input-output stream with one input and one output unit. Because the reservoir update in Equation 3 makes the state at time *t* depend on the input history through *u*_*t*−1_, the reservoir state **y**^(*t*)^ is paired with the target *s*_*t*_. The affine-linear readout is fitted to all post-washout reservoir states and targets using the same pseudoinverse method described below.

Independent training and test streams each contain 5,000 time steps. The first 200 time steps of each stream are used as washout and excluded from readout fitting and performance evaluation. The input-weight width is *w*_*I*_ = 0.3, and all NARMA-10 calculations use reservoirs with *N* = 50 neurons. Very rarely, the conventional recurrence can diverge numerically for a long random input record. We therefore reject sequences containing non-finite target values or |*s*_*t*_|>10 and deterministically draw a replacement from the same seeded random-number stream.

The final comparison scans 17 equally spaced balance values from *b* = −1 to *b* = +1 and uses 50 random seeds for each balance. For every seed, the calm reservoir, homogeneous reservoir, weak-row reservoir, and gain-controlled reservoir receive identical training and test streams. The homogeneous, weak-row, and gain-controlled conditions also originate from the same strongly coupled recurrent matrix, making their comparison paired.

### 2.7 Optimal readout layer using pseudoinverse

The optimal weights and biases of the readout layer can be efficiently computed with the method of the pseudoinverse, based on the sequence of reservoir states and the target output (Compare, for example, Section 3.4. in Cucchi et al., 2022). Following these ideas, we proceed as follows:

Let *Y* ∈ ℝ^(*E*−1) × *N*^ be the matrix of reservoir states directly after each input episode, where *E* is the total number of episodes and *N* is the number of reservoir neurons. Let *Z* ∈ ℝ^(*E*−1) × *K*^ be the matrix of target output states, where *K* is the number of output units.

To account for biases in the readout layer, a column of ones is appended to *Y*, resulting in the matrix $Y_{\text{bias}}\in {\mathbb{R}}^{\left(E-1\right)\times \left(N+1\right)}$:


$$Y_{\text{bias}}=\left[\begin{array}{cc} Y & {\text{1}}_{E-1} \end{array}\right]$$

where ${\text{1}}_{E-1}\in {\mathbb{R}}^{\left(E-1\right)\times 1}$ is a column vector of ones.

The weights and biases of the readout layer are computed by solving the following equation using the pseudoinverse of *Y*_bias_:


$$\begin{array}{l} W_{\text{bias}}=Y_{\text{bias}}^{+}Z \end{array}$$ (10)

where $Y_{\text{bias}}^{+}$ is the Moore-Penrose pseudoinverse of *Y*_bias_, and $W_{\text{bias}}\in {\mathbb{R}}^{\left(N+1\right)\times K}$ contains both the readout weights and the biases.

To compute the pseudoinverse, we first perform a singular value decomposition (SVD) of *Y*_bias_:


$$Y_{\text{bias}}=USV^{⊤}$$

where *U* ∈ ℝ^(*E*−1) × (*E*−1)^ is a unitary matrix, *S* ∈ ℝ^(*E*−1) × (*N*+1)^ is a diagonal matrix containing the singular values, and *V*^⊤^ ∈ ℝ^(*N*+1) × (*N*+1)^ is the transpose of a unitary matrix.

The pseudoinverse of *Y*_bias_ is computed as:


$$Y_{\text{bias}}^{+}=VS^{+}U^{⊤}$$

where *S*^+^ ∈ ℝ^(*N*+1) × (*E*−1)^ is the pseudoinverse of the diagonal matrix *S*. The pseudoinverse *S*^+^ is obtained by taking the reciprocal of all non-zero singular values in *S* and leaving zeros unchanged.

Finally, after inserting $Y_{\text{bias}}^{+}$ into Equation 10, the optimal readout weights *W* ∈ ℝ^*K*×*N*^ and biases *b* ∈ ℝ^*K*^ are extracted from the extended matrix *W*_bias_ as


$$W={\left(W_{\text{bias}}^{⊤}\right)}_{1:N,:}$$

$$b_{w}={\left(W_{\text{bias}}^{⊤}\right)}_{N+1,:}$$

where the first *N* rows of $W_{\text{bias}}^{⊤}$ define the readout weights and the last row defines the biases.

### 2.8 Fluctuation measure

The neural fluctuation measure *F* quantifies the average temporal variability of reservoir activations. For each neuron *n*, we compute the standard deviation σ_*n*_ of its activation time series $y_{n}^{\left(t\right)}$. The global fluctuation is defined as the mean over the standard deviations of the individual neurons:


$$F={\langle \sigma_{n}\rangle }_{n}$$

Since tanh-neurons produce outputs in [−1, +1], the fluctuation *F* lies in [0, 1]. A value of *F* = 0 indicates a resting or fixed point state, while *F* = 1 corresponds to perfect two-state oscillation (e.g., alternating between +1 and −1).

### 2.9 Covariance measure

To assess temporal covariances, we compute the average product of the activation of neuron *m* at time *t* and neuron *n* at time *t*+1:


$$C_{mn}={\langle y_{m}^{\left(t\right)}\cdot y_{n}^{\left(t+1\right)}\rangle }_{t}$$

Unlike the Pearson correlation coefficient, we deliberately avoid subtracting the mean or normalizing by the standard deviations. This ensures that the matrix elements *C*_*mn*_ remain well-defined even when one or both signals are constant, as in a fixed point state.

The global covariance measure is defined as the average over all neuron pairs, without differentiating between diagonal and off-diagonal elements:


$$C={\langle C_{mn}\rangle }_{mn}$$

Owing to the bounded output of the tanh neurons, the covariance values *C* always lie within the range [−1, +1].

### 2.10 Non-linearity measure

The shape of the activation distribution *p*(*y*) reflects whether the reservoir operates in a linear or non-linear regime. A central peak at *y* = 0 indicates a linear regime; two peaks near ±1 indicate saturation and thus non-linearity.

We define a non-linearity measure


$$N=f_{A}-f_{B}+f_{C}$$

based on the fractions of neural activations falling into the following intervals:


$$\begin{array}{l} f_{A}\in \left[-1,-0.5\right)\\ f_{B}\in \left[-0.5,+0.5\right]\\ f_{C}\in \left(+0.5,+1\right] \end{array}$$

The resulting measure *N* ∈ [−1, +1] distinguishes three regimes: *N* ≈ −1 for linear operation, *N* ≈ 0 for intermediate or flat activation, and *N* ≈ +1 for saturated, digital-like behavior.

This intuitive yet robust definition proved most effective among several tested alternatives. It captures the essential qualitative transition in *p*(*y*) from unimodal (linear) to bimodal (non-linear) distributions, as highlighted in earlier studies.

### 2.11 Accuracy measure

In the sequence generation task, we evaluate performance by comparing the actual output sequences *Z*_act_ of the readout layer with the corresponding target sequences *Z*_tar_, and compute the root-mean-square error *E*_RMS_. This error is normalized by the standard deviation Δ*Z*_tar_ of the target sequences.

To obtain an accuracy measure *A* ∈ [0, 1], we define:


$$A=\frac{1}{1+\left(E_{\text{RMS}}/\text{Δ}Z_{\text{tar}}\right)}$$

Note that *A* ≈ 0.5 when the RMS error is comparable to the variability of the target data, and *A* = 1 when the output *Z*_act_ matches the target *Z*_tar_ exactly.

In classification tasks, the accuracy *A* is simply defined as the fraction of correctly predicted class labels.

### 2.12 Robust performance measures

For both continuous-output tasks, the normalized root-mean-square error of an individual run is defined in Equation 11.


$$\begin{array}{l} \text{NRMSE}=\frac{E_{\text{RMS}}}{\text{Δ}Z_{\text{tar}}}. \end{array}$$ (11)

The normalization uses the standard deviation of the target values only. Consequently, a constant predictor equal to the target mean has NRMSE = 1, whereas a perfect prediction has NRMSE = 0. The accuracy defined above is equivalently *A* = 1/(1+NRMSE). Thus, *A* > 0.5 is equivalent to outperforming the constant target-mean predictor.

Because replicated reservoir calculations can have strongly skewed error distributions with rare severe failures, arithmetic means alone can obscure typical performance. For each balance and reservoir condition, we therefore additionally report the median of log_10_(NRMSE). Exact or numerically near-zero errors are replaced by 10^−12^ only for taking this logarithm. The accompanying interquartile range (IQR) extends from the 25th to the 75th percentile of the run-level log errors and summarizes the central half of the realizations.

Two threshold-based measures describe the tails of the distribution. The successful-run probability is the fraction *P*(NRMSE < 1) of realizations that outperform the target-mean baseline. The catastrophic-run probability is the fraction *P*(*A* < 0.1) of realizations with extremely poor accuracy; under the above transformation this corresponds to NRMSE>9. All probabilities and quantiles are computed from individual runs before any aggregation across random seeds.

**Figure 2:**
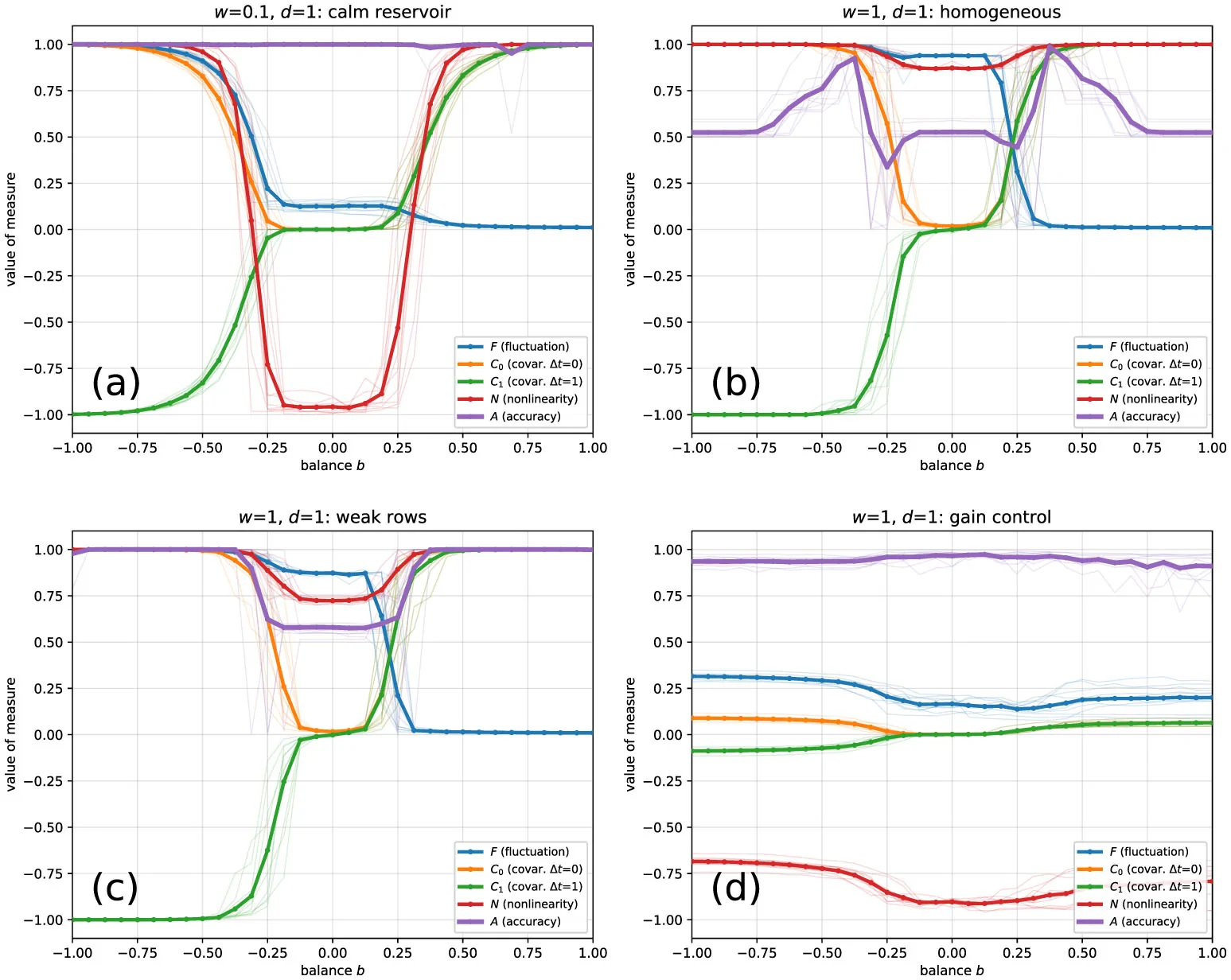
Replicated scans of dynamical properties and task accuracy over the excitatory/inhibitory balance parameter *b*. Thin semi-transparent curves show the results of ten individual network realizations for each quantity, using the same quantity-specific colors as the corresponding thick foreground curves. The thick curves and circular markers show arithmetic means over the ten realizations. The same set of random seeds is used throughout the balance scan, making the visible spread and shifts of sharp transition points a direct measure of realization dependence. **(a)** Non-structured system with the coupling strength reduced to *w* = 0.1. Even in this weakly coupled reservoir, three distinct dynamical regimes appear. In the globally oscillating regime at strongly negative *b*, the fluctuation *F* (blue), the non-linearity *N* (red), and the instantaneous covariance *C*_0_ (orange) approach the value +1, whereas *C*_1_ (green), the covariance at lag time one, approaches −1. In the calm regime around *b* = 0, fluctuations *F* are small, the non-linearity parameter *N* is close to −1, and both covariances are close to zero. In the global fixed-point regime at strongly positive *b*, *N*, *C*_0_, and *C*_1_ approach +1, whereas *F* becomes zero. Despite these regime changes, the mean accuracy *A* (purple) remains close to 1 across almost the complete balance range because of the weak coupling strength. **(b)** Non-structured system with the coupling strength increased to *w* = 1. Around *b* = 0, the calm state is replaced by high-amplitude chaotic spontaneous fluctuations, with small covariances and values close to one for *F* and *N*. Mean accuracy is close to chance level in the oscillatory, chaotic, and fixed-point regimes and improves only within narrow transitional regions near the two edges of chaos. The individual curves show that the locations and widths of these regions vary markedly among network realizations. **(c)** Structured system with 20 percent weak rows. The dynamical quantities remain qualitatively similar to **(b)**, although *F* and *N* are reduced in the chaotic regime. Accuracy is close to one throughout broad unbalanced regions but remains only slightly above chance level in the central chaotic regime. The transition boundaries again display substantial realization dependence. **(d)** System with automatic gain control. The non-linearity *N* remains relatively close to −1, fluctuations *F* are moderate and mainly input-driven, and both covariances remain small. The regulated dynamics therefore resemble the calm regime of the weakly coupled reservoir over almost the complete balance range. Accuracy remains high and shows considerably less realization-to-realization variation than in the homogeneous and weak-row conditions.

For continuity with the original presentation, Figure 2 retains arithmetic means and displays every individual realization instead of mean ± standard deviation. The added error analyses use medians and IQRs because their run-level distributions can be highly skewed and heavy tailed; in such cases a symmetric standard-deviation band around the mean is not a faithful description of either typical performance or failure risk. Seed counts and pairing are stated for each experiment in the corresponding Results paragraph or Supplementary material.

## 3 Results

### 3.1 Naming conventions and essential methods

In the following section, we briefly introduce the naming conventions as well as the tools for control, visualization, and analysis that are used throughout the paper. Further details are provided in the Methods section.

#### 3.1.1 Framework

We consider a reservoir computer (RC) consisting of an input matrix, a Recurrent Neural Network (RNN) serving as the reservoir, and a readout layer trained using the pseudoinverse method. To make multiple runs feasible for many parameter combinations, we restrict ourselves to reservoirs with only 50 neurons. This choice also allows us to draw on experience from our previous publications, where the same reservoir size was mostly used.

#### 3.1.2 Statistical control parameters

The elements *w*_*ij*_ of the weight matrix describing the recurrent connections among the reservoir neurons are random, but obey three statistical control parameters. The density *d* ∈ [0, 1] denotes the fraction of non-zero elements. The main experiments use fully connected networks with *d* = 1; sparse networks are evaluated only as a Supplemental control. The coupling strength *w* is defined as the standard deviation of the normally distributed weight matrix elements (in a balanced system). The balance *b* ∈ [−1, +1] characterizes the relative predominance of excitatory and inhibitory connections: *b* = −1 corresponds to exclusively negative weights, *b* = 0 to a balanced system, and *b* = +1 to exclusively positive weights. A reservoir described by these three parameters and without further modification is called homogeneous, because all matrix elements follow the same statistical rule.

#### 3.1.3 Weight matrix structuring

**Figure 3:**
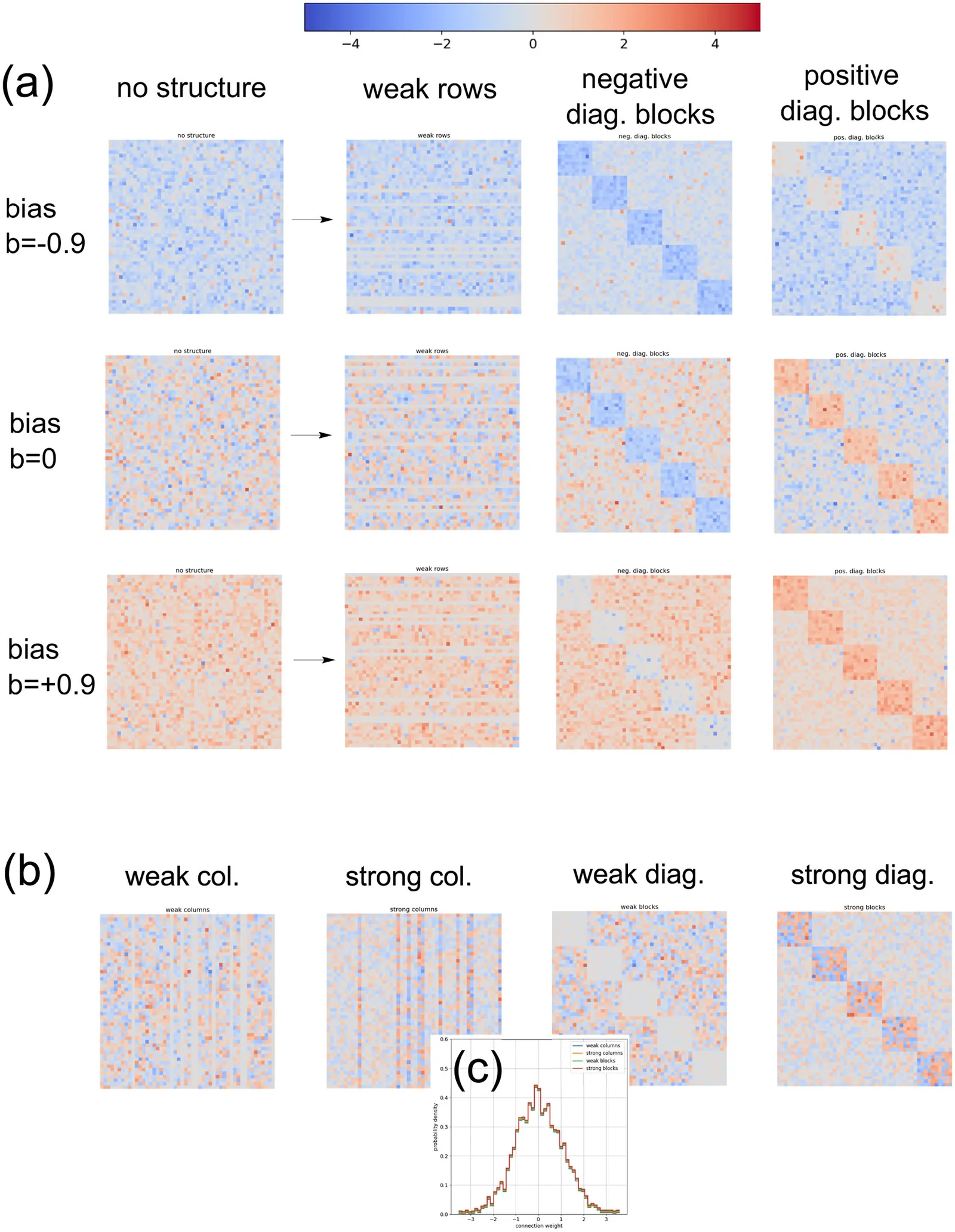
Structuring of the weight matrix. **(a)** Three examples of GP-enhancing types of permutative matrix structuring, shown for three different balance values *b*. In each case, the full reservoir connection matrix is displayed with color-coded weights. **(b)** Four examples of permutative matrix structuring that do not lead to an improvement in global performance. **(c)** Probability distributions of connection weights for four different types of permutative matrix structuring. The distributions remain unchanged. The four curves have been slightly shifted vertically to improve visibility.

Without changing its global weight distribution *p*(*w*), a homogeneous weight matrix can be structured by redistributing its elements across the matrix in various semi-regular ways. For example, a geometrically defined subset of the matrix, such as a small random fraction *f*_1_ of all matrix rows, may preferentially receive more extreme elements from the distribution *p*(*w*), such as those with the weakest or strongest magnitudes, or those with the most negative or most positive values. Instead of selecting random rows as the *f*_1_ subset, one can also use columns or quadratic blocks of size *S* along the matrix diagonal. We refer to this distribution-conserving method of patterning the weight matrix as “Permutative Structuring.” Some examples of homogeneous and structured matrices are shown in Figure 3.

#### 3.1.4 Neural activity visualization

**Figure 4:**
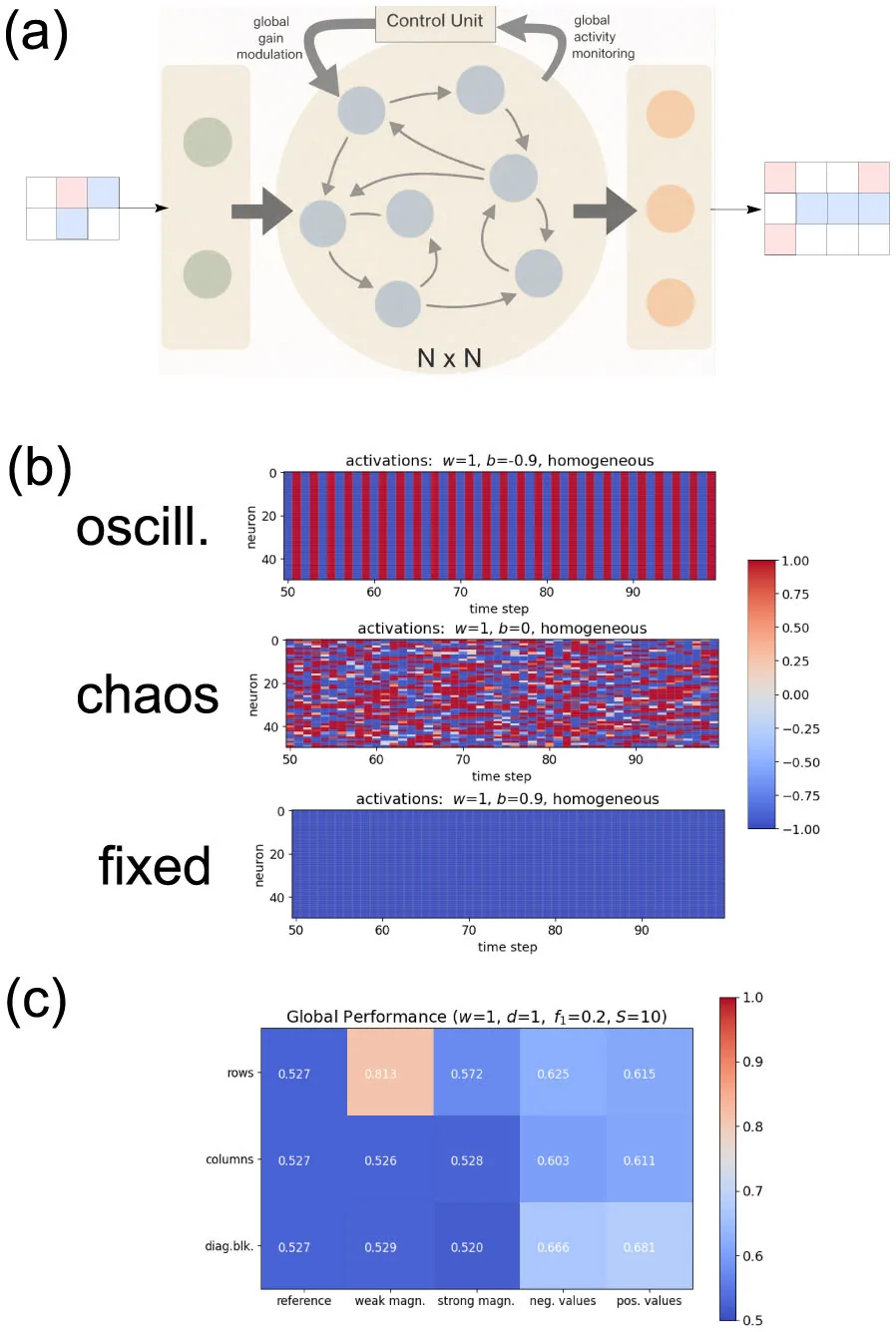
Overview. **(a)** Reservoir computer consisting of a recurrent neural network (RNN with *N* neurons, central circle with blue dots), an input layer (left box with green dots), and a readout layer (right box with orange dots). A control unit for dynamic gain regulation can optionally be placed on top of the RNN. The colored grids symbolize sequences of input and output vectors. **(b)** Three direct plots of neural activations (color coded) over time in a densely connected RNN of 50 neurons with tanh activation functions. Because the coupling strength is large (*w* = 1), the system develops global oscillations for strongly negative balance parameters such as *b* = −0.9, chaotic fluctuations for highly balanced systems such as *b* = 0, and global fixed points for strongly positive balance parameters such as *b* = +0.9. All these attractor regimes are detrimental to task-related information processing. **(c)** The Global Performance (GP, color coded) measure, defined as the accuracy averaged over nine bias values spanning the full range from −1 to +1, evaluated for 3 × 5 different types of permutative matrix structuring that all preserve the global weight distribution (compare main text and Figure 3). The best GP is obtained when 20 percent of the rows in the weight matrix (representing neural inputs) have a weaker-than-average magnitude.

The dynamical processes in the reservoir can be most directly visualized by plotting the individual activations of all neurons over successive time steps (compare Figure 4b). Since the neurons use tanh activation functions, their continuous outputs are always in the range from −1 to +1. However, when the system develops strong spontaneous dynamics and runaway excitations drive the neurons deep into saturation, it becomes impossible to see the very small input-related perturbations that may still be superimposed on the spontaneous activity and that could nevertheless be exploited by the readout layer.

#### 3.1.5 Principal component analysis

**Figure 5:**
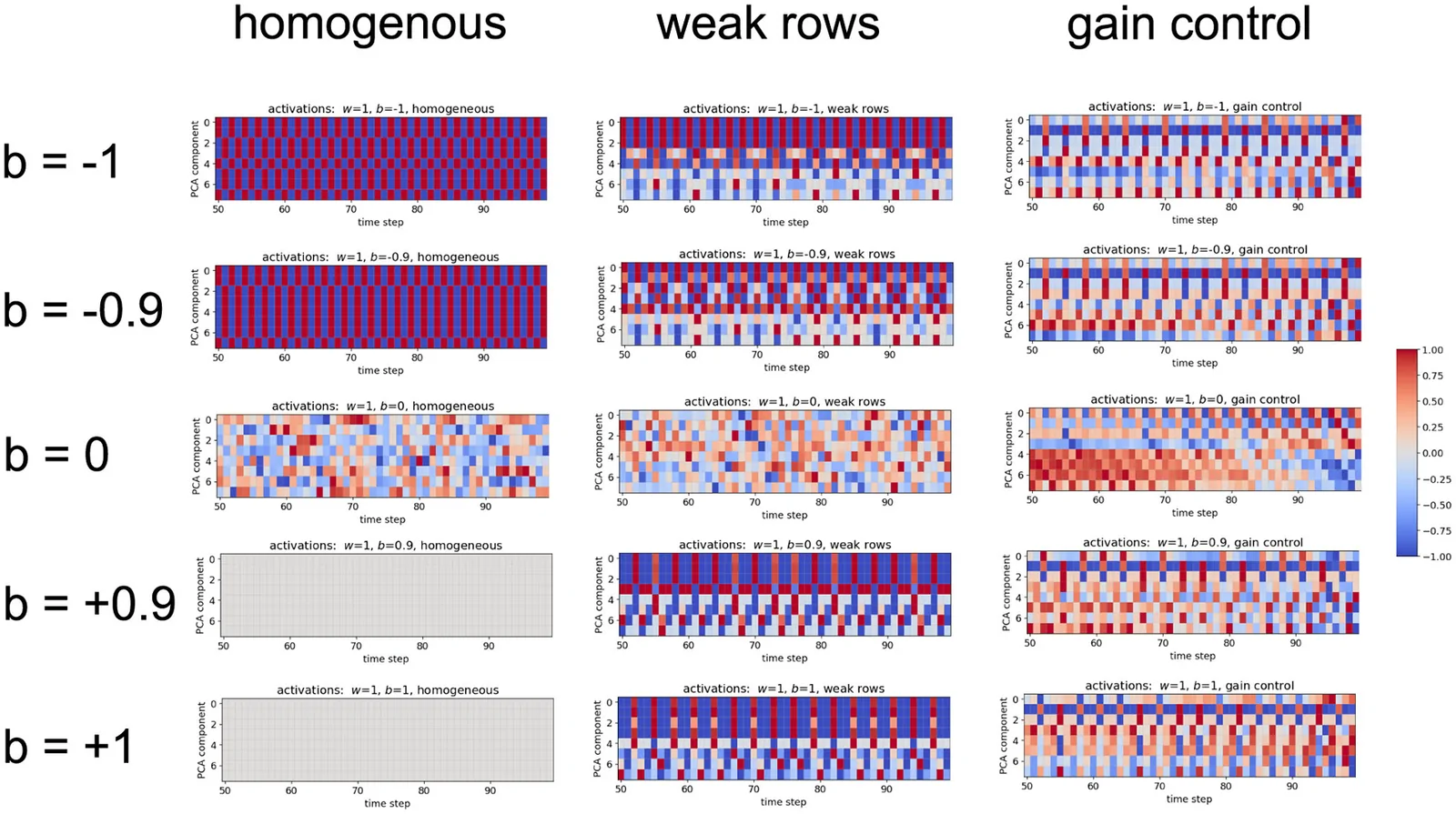
Principal Component Analysis (PCA) of reservoir activations. Each of the 5 × 3 plots represents the lowest eight principal components of the reservoir activation states as a function of time. The magnitudes of these components differ greatly. Since the readout layer is insensitive to absolute magnitude, we normalize the values within the observation window to the full range from −1 to +1 to improve visibility. The first column of plots shows the homogeneous (non-structured) system. In the range of strongly negative bias (*b* = −1 and *b* = −0.9), all PCA components reflect the reservoir's global oscillations with a period of two. In the balanced case (*b* = 0), the components fluctuate chaotically. In the range of strongly positive bias (*b* = +0.9 and *b* = +1), where the reservoir resides in a global fixed-point attractor, all PCA components are zero because the mean is subtracted in the PCA analysis. The second column of plots shows the structured system with some rows of reduced magnitude in the weight matrix. In the balanced case (*b* = 0), we again observe chaotic behavior. However, in all strongly unbalanced cases (*b* = −1, *b* = −0.9, *b* = +0.9 and *b* = +1), the PCA components exhibit much richer dynamics. Some display a quasi-periodic pattern with a period of three, corresponding to the length of each input episode. These components therefore encode input-related information that can be exploited by the readout layer. The third column of plots shows the system equipped with a unit for automatic gain control. Here, the information encoded in the PCA components is even more diverse and more strongly aligned with the pulses of input.

For this reason, we also perform a Principal Component Analysis (PCA) on the entire temporal evolution of the reservoir states and then plot the dominant PCA components as a function of time (compare Figure 5). The different PCA components can vary in magnitude by many orders of magnitude. Since the readout layer is insensitive to these differences, we normalize all components individually within the displayed time window to the range from −1 to +1. This normalization makes even weak patterns visible that are clearly related to the input signals (rather than to the spontaneous dynamics), because they change in synchrony with the pulses of the input episodes.

#### 3.1.6 Dynamical measures

For a more quantitative analysis, we compute four aggregating dynamical measures from the entire, multi-episode temporal evolution of the reservoir states: The fluctuation *F* ∈ [0, 1] quantifies the global level of change in the neural activations, averaged both over the neural population and over time. Two covariances *C*_0_ and *C*_1_, both ranging from −1 to +1, measure the (non-normalized) degree of correlation between neurons, averaged over all neuron pairs. Here, *C*_0_ denotes the instantaneous correlation and *C*_1_ the correlation at lag time one. Finally, the non-linearity *N*, ranging from −1 to +1, quantifies whether the neurons operate predominantly in the linear or in the non-linear, saturated part of their sigmoidal activation function. Values close to −1 indicate linear behavior, whereas values close to +1 indicate saturated behavior. Together, these four measures allow us to classify the reservoir's dynamical state into four broad regimes, which we refer to as “oscillatory,” “chaotic,” “calm,” and “fixed point.” It is particularly instructive to plot these measures as functions of some control parameter that is swept through its range and to observe how the RNN is switching between dynamical regimes accordingly (compare Figure 2).

#### 3.1.7 Task and accuracy

We are primarily interested in the question of how structural properties of the weight matrix (or regulating control mechanisms) influence the dynamical regime of the RNN and which of these regimes are suitable for task-related information processing. The quality of information processing, in turn, is most directly assessed through the accuracy achieved in specific tasks. This poses a challenge, however, because it is nearly impossible to take into account all possible types of tasks with their different information-processing requirements. The main text therefore uses two complementary benchmarks. The original Sequence Generation task is intentionally short and strongly input driven: the RC receives class-specific input vectors and must produce predefined two-step output sequences. It directly tests whether recent input remains discernible despite spontaneous reservoir dynamics, but places only limited demands on long-term memory. NARMA-10 adds a standard non-linear temporal prediction problem with a ten-step dependency. The components of both input and output vectors in Sequence Generation are continuous numbers between −1 and +1. Although this task may not be particularly useful for real-world applications, it provides a clear test of whether the reservoir dynamics are mainly determined by the most recent input sequence, by remnants of earlier input episodes, or by spontaneous dynamics with the potential for runaway excitation. To assess performance in the task, we first compute the root-mean-squared error between the target and the actual output vectors and then convert this error into an accuracy *A*. This accuracy is approximately 0.5 at chance level and approaches 1 when performance is near perfect (compare purple curves in Figure 2). Conclusions shared by both benchmarks are more persuasive than conclusions from Sequence Generation alone, but two tasks still do not justify claims of universal task generality.

### 3.2 Problem of runaway excitation

#### 3.2.1 Weakly coupled reservoirs

It is instructive to first consider a reservoir with relatively weak recurrent connections, such as in the case of a coupling strength *w* = 0.1 (compare Figure 2a). In this situation, the dynamical regime of the RNN depends on the balance parameter *b*.

The revised balance scans in Figure 2 are based on 10 independent network realizations for every condition and balance value. Thick curves denote arithmetic means, whereas thin semi-transparent curves show the individual realizations. Unless stated otherwise, the following description refers to the mean curves; the spread of individual curves indicates realization dependence.

When *b* is close to zero, corresponding to a balanced weight matrix with equal amounts of negative and positive entries, the weakly coupled RNN operates in a calm regime. This regime is characterized by vanishing covariances *C*_0_, *C*_1_, small fluctuations *F*, and a non-linearity parameter *N* close to −1, indicating that neurons operate mainly in the linear regime. The dynamics are primarily determined by the most recent input, there is no spontaneous activity, and the mean accuracy *A* is nearly perfect.

However, when the balance is tuned too far into the positive range, so that most connections become excitatory, herd effects emerge and positive feedback drives the system into a global fixed-point attractor. In this regime, fluctuations *F* nearly vanish, both covariances approach +1, and all neurons are driven into saturation, indicated by a non-linearity parameter *N* approaching +1. In general, such saturated, locked-in neurons are not well-suited for information processing, because the trajectory of the reservoir state should be able to reflect changes in the input signals. Nevertheless, in the weakly coupled RNN, tiny input-related differences in neural activations remain present even in the saturated neurons. The readout layer can still exploit these differences and, remarkably, mean accuracy *A* remains close to perfect even in the fixed-point regime, although isolated realizations show performance dips near sharp dynamical transitions.

Another herd effect occurs when the balance is tuned too far into the negative range, so that most connections become inhibitory. In this case, negative feedback drives the system into global oscillations with period two, in which all neurons alternately switch between negatively and positively saturated states. Again, this situation would normally be detrimental for information processing, but due to the weak coupling mean accuracy remains close to perfect even in this oscillatory regime.

#### 3.2.2 Strongly coupled reservoirs

The ability to perform input-related information processing collapses almost completely when the coupling strength is increased to *w* = 1 (compare Figure 2b).

Even when the balance *b* is around zero, the RNN is no longer calm. Instead, the strong recurrent bipolar connections lead to spontaneous chaotic fluctuations (compare Figure 4b, middle plot), which are not related to the momentary external input. In this chaotic regime, both covariances *C*_0_ and *C*_1_ are close to zero, whereas the fluctuation *F* and the non-linearity *N* approach the upper limit +1. As a consequence, mean accuracy *A* drops to approximately chance level 0.5.

In the vicinity of *b* = −1 and *b* = +1, we again observe saturating oscillatory or fixed-point behavior (compare Figure 4b, upper and lower plot), respectively. In contrast to the weakly coupled RNN, however, mean accuracy now remains close to chance level also in these highly unbalanced systems.

This leaves the two regions between chaos and oscillatory behavior, as well as between chaotic and fixed-point behavior, as the only transitional regimes in which mean accuracy *A* can rise to satisfactory levels. The individual curves show, however, that the positions and widths of these favorable windows vary markedly among network realizations. The result therefore supports the view that transitions near the edge of chaos can enable useful information processing, but it does not define a unique, realization-independent optimum. In practical applications, exploiting such narrow windows would require careful and network-specific tuning of the balance parameter.

Note that in the vicinity of *b* = −1 and *b* = +1, the four dynamical measures *F*, *C*_0_, *C*_1_, and *N* have very similar values in the weakly and strongly coupled RNN (compare Figures 2a, b). Direct plots of the neural activations also appear identical (as in the upper and lower plots of Figure 4b) in strongly unbalanced systems, both in the weakly and strongly coupled case. Yet the accuracy *A* reaches perfection in one case and remains at chance level in the other. This clearly indicates that our dynamical measures and direct activation plots are not sensitive enough to reveal the presence or absence of tiny input-related perturbations superimposed on the global herd behavior. For this reason, we use in the following sections Principal Component Analysis with visual magnitude enhancement to reveal these subtle effects.

#### 3.2.3 Statement of the problem

We are now in a position to formulate the problem and research goal addressed in this work: In strongly coupled RNNs, herd effects prevent information processing both in strongly unbalanced systems and in the perfectly balanced system. How can this limitation be overcome without fine-tuning the balance parameter to the narrow sweet spot at the edges of chaos?

### 3.3 Permutative structuring of the weight matrix

#### 3.3.1 Previous work

In a previous publication (Metzner et al., 2025b) we demonstrated that Dale's principle (requiring that every output column in the weight matrix should be either strictly positive or strictly negative) has a small performance-enhancing effect, whereas the reciprocal symmetry of Hopfield networks (requiring that *w*_*ij*_ should be similar to *w*_*ji*_) generally tends to degrade performance.

#### 3.3.2 Leptokurtic weight distributions

In the same paper we also observed a modest performance gain when introducing quadratic patches into the weight matrix, some of which contained larger-than-average magnitudes and others smaller-than-average magnitudes. Although the overall standard deviation was strictly conserved in that investigation, the patch-like patterning made the overall distribution more leptokurtic, thereby increasing the fraction of near-zero weights. As it turned out after publication, this effective reduction of the connection density *d* was mainly responsible for the observed performance enhancement.

#### 3.3.3 Improved patterning method

For this reason, we have now developed a new method of matrix patterning that strictly conserves the entire weight distribution, because it is based on a controlled permutation of the existing weights across the matrix (see Methods section for details).

#### 3.3.4 Comparing types of structuring

We apply four different kinds of modifications (weaker-than-average magnitudes, stronger magnitudes, more negative values, and more positive values) to a small subset of the matrix elements, arranged in three different geometric patterns (rows, columns, and diagonal blocks), thus creating twelve different combinations of permutative structuring. For each combination, we compute a global performance measure GP, defined as the accuracy averaged over nine equally spaced balance values *b* in the range from −1 to +1. The baseline is the GP obtained for the non-structured, homogeneous matrix, which is 0.527. The results are summarized in Figure 4c.

#### 3.3.5 Weak rows

We find the strongest performance enhancement (raising the GP from 0.527 to 0.813) when weaker-than-average weight magnitudes are assigned to 20 percent of randomly selected rows in the weight matrix. Consequently, the remaining 80 percent of rows contain larger-than-average magnitudes. Since the matrix rows represent the input weights of the neurons, this best-performing RNN contains a subpopulation of 20 percent of neurons that receive particularly weak inputs from the rest of the network. In situations where the network suffers from herd effects that generate runaway excitations and lead to performance-degrading saturation, this subpopulation can remain partially decoupled from the global network dynamics and still provide useful input-related information to the readout layer.

#### 3.3.6 Unipolar blocks

The next best performance enhancement (raising the GP from 0.527 to 0.666 or 0.681, respectively) is observed when all defined diagonal blocks of linear size 10 receive preferentially negative or preferentially positive values. A positive block, for example, corresponds to a kind of module with predominantly excitatory internal connections that is embedded in a larger surrounding network with the opposite polarity of connections.

#### 3.3.7 Overview and visualization

Of the 12 investigated combinations of matrix structuring, eight lead to an enhancement of the GP. The top three combinations are shown for different bias values *b* in Figure 3a. The four combinations that do not increase performance are shown in Figure 3b.

### 3.4 Effect of weak rows

We next investigate the effect that a small subset of neurons with weak input weights has on the dynamics of the reservoir and how this influences its information-processing capability.

#### 3.4.1 Dynamics as a function of balance

For this purpose, we again scan the balance parameter *b* over its entire range and compute the four dynamical measures as functions of *b* (Figure 2c). Compared with the corresponding homogeneous system (Figure 2b), the mean quantities *F*, *C*_0_, *C*_1_, and *N* exhibit very similar behavior. Only in the chaotic regime around *b* = 0 do the weak rows substantially reduce the fluctuation *F* and the non-linearity *N*, which is accompanied by an increase of mean accuracy *A* to slightly above chance level. In strongly unbalanced regimes close to *b* = −1 and *b* = +1, weak rows allow mean accuracy to approach the perfect level of 1 even though little difference is visible in the four mean dynamical measures. The individual curves additionally reveal considerable realization dependence near the transition boundaries. These results confirm that the four dynamical measures characterize the broad reservoir regime but do not by themselves quantify its information-processing capability.

#### 3.4.2 PCA-analysis of the weak row effect

To gain further insight into the effect of weak rows, we next compare the time dependence of the reservoir's lowest PCA components with those of the corresponding homogeneous system (Figure 5, left and middle columns of plots). Here, we are mainly interested in the balance regions that are unsuitable for information processing in the homogeneous system. We therefore restrict this PCA analysis to five representative balance values *b* ∈ {−1, −0.9, 0, +0.9, +1}.

#### 3.4.3 PCA-components in the homogeneous RNN

In the homogeneous RNN at *b* = −1 and *b* = −0.9, we observe the typical period-two global oscillation in all of the lowest eight PCA components (Figure 5, left column, upper two plots). Recall that the time series of each component is separately rescaled to the range from −1 to +1 for better visibility. Nevertheless, the plot reveals that the values within each time series flip in a strictly regular way between only two binary states and therefore do not provide the input-dependent differences required by the readout layer.

In the balanced case at *b* = 0, all components exhibit chaotic behavior (left column, middle plot). These fluctuations are likewise not sufficiently determined by the input signals.

Finally, at *b* = +0.9 and *b* = +1, we have seen before that the RNN resides in a global fixed point, in which all neuron activations remain at the same constant, highly saturated value. Since this constant activation vector—corresponding to the mean or “center point” of the high-dimensional “cloud” of momentary activation vectors—is subtracted in the PCA analysis, all components take the value zero (left column, lower two plots). Clearly, this again provides no useful information for the readout layer.

#### 3.4.4 PCA-components in the RNN with weak rows

The introduction of a few weak rows in the weight matrix does not prevent the PCA components from appearing chaotic in the perfectly balanced system at *b* = 0 (Figure 5, middle column, center plot). Nevertheless, recall that even in this chaotic regime the accuracy is now slightly above chance level, meaning that some input-related information must be hidden within these irregular fluctuations of the PCA components.

The situation changes much more dramatically for the other four considered balance values (middle column, upper and lower plots). The momentary values of the PCA components are no longer restricted to only three possible values (−1, 0, and +1, after rescaling). At least some of the higher components can now choose from a broader and more informative domain of values. Moreover, in some components one can observe a quasi-periodic, regular pulsing with a period of three, corresponding to the length of each input episode. Closer inspection reveals two broad categories of pulses, reflecting the two classes of input vectors in the Sequence Generation task. Even within pulses of the same category, however, the values differ slightly. This richer, input-related information content in the PCA components enables the readout layer to produce the target output sequences with high accuracy, even when the system is globally in an oscillatory or fixed-point regime.

### 3.5 Effect of automatic gain control (AGC)

#### 3.5.1 Motivation of AGC

As we have seen, suitable patterning of the weight matrix, without altering the distribution of its elements, can make a strongly coupled reservoir capable of information processing even in cases of large imbalance between excitatory and inhibitory connections. In the chaotic regime, the patterning also slightly increases the accuracy to values above chance level. Nevertheless, in a practical setting one would still attempt to keep the balance away from the chaotic regime in order to maximize information-processing performance. We therefore seek additional extensions of the reservoir computer that make its performance less dependent on the detailed statistical and structural properties of the weight matrix.

#### 3.5.2 Inspiration by the brain

In particular, we consider an external control unit that continuously monitors the dynamical state of the reservoir and, if necessary, sends back regulatory feedback signals in order to keep the network dynamics close to a desired target state (Figure 4a, top). Inspired by biological neural systems, where the overall excitability and effective coupling strength of neurons are modulated by neuromodulators, hormones, or glial interactions, we allow only global (not neuron-specific) signals to be exchanged between the control unit and the reservoir of neurons.

#### 3.5.3 Implementation of AGC

As described in more detail in the Methods section, we aim to regulate the global activity of the reservoir, quantified as a running time average of the momentary root-mean-squared activation across all neurons, to a predefined target value. To allow the control unit to influence this global activity, we do not modify the entries of the weight matrix individually, but instead modulate a single global pre-factor of that matrix according to the deviation between the actual and the target activation. This mechanism can also be interpreted as a dynamical regulation of the effective coupling strength *w*.

#### 3.5.4 Dynamics and performance with AGC

We apply this method of AGC to the strongly coupled system with *w* = 1 and again scan the balance parameter *b* across its entire domain (Figure 2d). We find that all four dynamical parameters *F*, *C*_0_, *C*_1_, and *N* now exhibit behavior that qualitatively resembles that of the weakly coupled (*w* = 0.1) reservoir in the calm (*b* = 0) regime, but now across the entire range of balance values *b*. Mean fluctuations *F* remain within a controlled moderate range rather than being fixed exactly at the activity target, and the individual curves show only limited variation over most balances. The non-linearity *N* remains close to −1, indicating that neurons operate mainly in a linear or only weakly non-linear region of the activation function. Both covariances *C*_0_ and *C*_1_ remain small in magnitude, indicating that both oscillatory and fixed-point behavior are strongly suppressed by the continuous dynamical regulation of reservoir activity. Most importantly, mean accuracy *A* remains high over the complete balance range and varies much less across realizations than in the homogeneous or weak-row conditions, although the spread increases somewhat toward strongly positive balance. A visualization of the time-dependent PCA components (Figure 5, right column) reveals that the reservoir now produces a set of diverse states that appear even richer in information than those of the system with weak rows (Figure 5, middle column). Again, many of the PCA components show the pulsing behavior with period three, indicating the representation of input-related content. This remains true even in the balanced system at (*b* = 0), because AGC suppresses the chaotic regime.

### 3.6 Alternative performance measures

**Figure 6:**
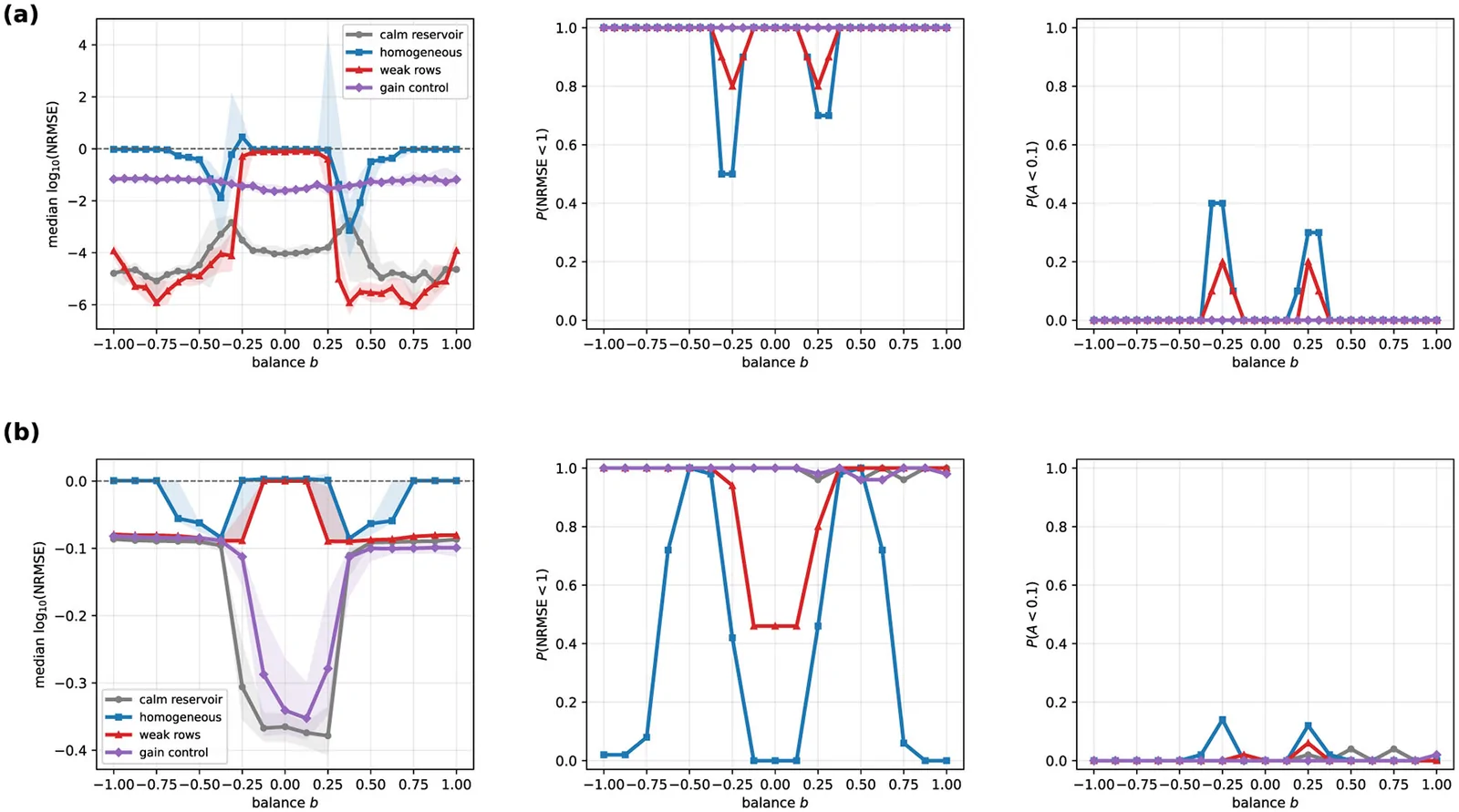
Robust performance across random reservoir realizations for two continuous-output tasks. Row **(a)** shows the sequence-generation task evaluated over 10 random seeds at each of 33 balance values; row **(b)** shows NARMA-10 evaluated over 50 random seeds at each of 17 balance values. Gray, blue, red, and purple curves represent the calm reservoir, homogeneous strongly coupled reservoir, reservoir with weak rows, and reservoir with automatic gain control, respectively. The left column shows the median log_10_(NRMSE); shaded regions indicate the interquartile range from the 25th to the 75th percentile, and the dashed horizontal line marks NRMSE = 1. The center column shows the empirical fraction of successful runs, *P*(NRMSE < 1), which outperform the constant target-mean predictor. The right column shows the empirical fraction of catastrophic runs, *P*(*A* < 0.1), equivalently *P*(NRMSE>9). All finite runs, including catastrophic realizations, are retained in these statistics.

The accuracy transformation *A* = 1/(1+NRMSE) used above is intuitive but strongly compresses differences among good predictions near *A* = 1. Moreover, individual-run errors are strongly skewed in some balance regimes because a small number of reservoir realizations fail severely. We therefore complement mean accuracy with three distribution-oriented measures (Figure 6): the median log_10_(NRMSE) and its interquartile range quantify typical performance, *P*(NRMSE < 1) gives the fraction of runs that outperform the constant target-mean predictor, and *P*(*A* < 0.1) quantifies the risk of catastrophic failure. Importantly, finite catastrophic runs are retained rather than discarded as outliers.

For the sequence-generation task (Figure 6a), both the calm reservoir and AGC outperform the target-mean baseline in nearly every realization across the entire balance range, and neither condition produces a catastrophic run. The homogeneous strongly coupled reservoir is much less reliable in the transition regions between its principal dynamical regimes: the successful-run fraction falls to approximately 0.5–0.7 at several sampled balances, while the catastrophic-run fraction reaches approximately 0.3–0.4. Weak rows markedly improve reliability relative to the homogeneous reservoir, although a narrower failure region remains and the catastrophic fraction reaches approximately 0.2 at its worst sampled balances. The logarithmic error representation additionally reveals that weak rows achieve extremely small typical errors in broad strongly unbalanced regimes, whereas AGC maintains a somewhat higher but much more uniform error level over balance. Because only ten realizations are available for this task, the displayed success and catastrophe frequencies occur in increments of 0.1.

NARMA-10 provides a more demanding test because it requires non-linear temporal processing and memory. At balanced connectivity, mean accuracy is 0.698 for the calm reservoir and 0.673 for AGC, compared with 0.498 for the homogeneous and 0.500 for the weak-row reservoir (see also the individual and mean accuracy curves in Supplementary Figure S2). The robust measures in Figure 6b sharpen this comparison. The calm reservoir gives the smallest typical error near balanced connectivity, while AGC approaches its performance and succeeds for almost every seed across nearly the complete balance range. By contrast, the homogeneous reservoir has only a narrow, balance-dependent success region; around *b* = 0, none of its realizations outperform the target-mean baseline. Weak rows substantially increase the successful-run fraction over much of the range, but near balanced connectivity only approximately 45 percent of realizations succeed. Catastrophic NARMA-10 failures are rare for all conditions, with the lowest observed risk under AGC. Thus, gain regulation does not eliminate the non-linear temporal-processing capability required for NARMA-10, but instead preserves most of the performance of the calm reference while making it far less sensitive to connectivity balance.

### 3.7 Optimal weak row fraction

The choice of 20 percent weak rows was initially motivated by exploratory calculations rather than by a systematic optimization. We therefore varied the weak-row fraction from 0 to 0.5 at five representative balance values, using 30 paired random realizations per fraction and balance (Figure 1a). For each balance-seed pair, all fractions use identical training and test data and the same initial homogeneous matrix; the selected weak-row sets are nested as the fraction increases. The fraction-zero condition is the unmodified homogeneous reservoir.

In the strongly inhibitory and excitatory regimes, *b* = −0.50 and *b* = +0.50, introducing an intermediate fraction of weak rows improves the median normalized error by several orders of magnitude. The best sampled median occurs at a fraction of 0.24 for *b* = −0.50 and 0.16 for *b* = +0.50, but both curves exhibit a broad high-performance region that includes the originally selected value 0.20. Performance deteriorates again at very large fractions, showing that weakening an excessive part of the network is not advantageous.

The dependence is less pronounced or less reliable in the remaining regimes. At *b* = 0, the homogeneous median accuracy is approximately 0.514, rises to a sampled maximum of 0.556 at fraction 0.08, and remains similar at 0.547 for fraction 0.20. At the transition balances *b* = −0.25 and *b* = +0.25, the interquartile ranges are very broad, demonstrating strong realization dependence. The best sampled median accuracies are approximately 0.586 at fraction 0.12 for *b* = −0.25 and 0.578 at fraction 0.20 for *b* = +0.25.

No single fraction is therefore optimal for every balance or summary statistic. Averaged over the five balances, the successful-run probability is maximal at fraction 0.12, with a value of 0.853, while fraction 0.20 yields a nearly identical value of 0.840. The aggregate median log-error is smallest at fraction 0.24. We therefore interpret 20 percent not as a uniquely tuned optimum, but as a representative robust compromise within a broad intermediate-fraction range. This range strongly stabilizes unbalanced reservoirs, whereas changing the fraction provides only modest benefits in the chaotic balanced regime and cannot remove the large realization dependence near dynamical transitions.

For a new application, the weak-row fraction should therefore be treated as a validation parameter. A practical procedure is to evaluate a modest fraction grid at balances representative of the expected operating range, summarize performance using the median log_10_(NRMSE) and/or the successful-run probability, and choose a value from a broad performance plateau rather than the numerically best single point. The preferred fraction can depend on the task, coupling strength, reservoir size, balance distribution, and other connection statistics; 20 percent is a transparent default for the present setup, not a universal prescription.

### 3.8 AGC parameter studies

Finally, we examine whether the performance of AGC depends on fine-tuning its three control parameters. We vary the activity setpoint α, averaging factor μ, and feedback strength ϵ one at a time while holding the other two parameters at their standard values, α = 0.25, μ = 0.10, and ϵ = 0.25 (Figure 1b). Each scan uses the sequence-generation task, the same five representative balance values as the weak-row ablation, and 30 paired reservoir realizations per parameter value.

The activity setpoint has a clear lower threshold. At α = 0.05 and 0.10, the controller suppresses reservoir activity so strongly that median errors exceed the target-mean baseline by several orders of magnitude at all five balances. Performance improves abruptly between approximately α = 0.15 and 0.20. From α ≈ 0.20 to the largest tested value 0.60, all balances lie in a broad high-performance region containing the standard value α = 0.25. Thus, the setpoint must be sufficiently large to preserve task-relevant state variation, but it does not require narrow tuning once this threshold is crossed.

Increasing the averaging factor μ generally reduces the median error over the investigated range from 0.01 to 0.80. A small μ produces a slowly adapting activity estimate, whereas larger values allow the controller to track activity changes more rapidly. The lowest aggregate sampled error occurs at μ = 0.80, but the standard value μ = 0.10 already stabilizes all investigated balances. The monotonic improvement in this task does not establish that the fastest tested activity estimate is universally optimal, since larger μ may respond more strongly to temporal noise or behave differently for other tasks.

The feedback-strength scan confirms that active regulation is essential. With ϵ = 0, corresponding to the unregulated control, several balance regimes perform at or below the target-mean baseline and show large uncertainty. Even weak positive feedback causes a pronounced improvement, and a broad range of positive ϵ values performs well. The aggregate sampled optimum occurs near ϵ = 0.15, while the standard value ϵ = 0.25 gives comparable robust performance. Together, the three scans show that AGC does not rely on a narrowly tuned parameter triplet: very small α and absent feedback fail, but the standard values lie within broad effective regions. Because these are one-at-a-time scans, they characterize the marginal role of each parameter but do not exclude interactions among α, μ, and ϵ.

## 4 Conclusions

### 4.1 Summary

In the context of reservoir computing (RC), we address in this paper the problem of runaway excitations in recurrent neural networks (RNNs). These excitations arise from herd effects, particularly in dense neural networks with unbalanced excitatory and inhibitory connections. As a result, the network is driven into global oscillatory or fixed-point attractors in which all neurons are pushed into the flat, saturating region of their sigmoidal activation functions—an operating regime that is largely insensitive to external input signals.

As long as the overall coupling strength of the neural network remains sufficiently small, tiny input-related perturbations can still “ride on top” of these high-amplitude global attractors, allowing the readout layer to exploit these perturbations for the task at hand. However, in strongly coupled networks the neurons are driven so deeply into saturation that processing of input-related information becomes impossible, and the performance of the reservoir computer degrades drastically.

A related problem also emerges in balanced systems when the coupling strength becomes too large. In weakly coupled balanced RNNs, the dynamics is calm, quasi-linear, and primarily driven by external input, which is ideal for many information-processing tasks. In contrast, strongly coupled balanced RNNs transition from this calm regime into a chaotic regime that is also detrimental to input-related information processing.

Consequently, in strongly coupled reservoir computers the performance (for example in a task such as sequence generation), when plotted as a function of the balance parameter, remains close to chance level across most of the parameter range. Only two narrow peaks appear at the so-called “edges of chaos,” making practical operation difficult because it requires careful fine-tuning of the network parameters.

As a first countermeasure to these runaway excitations, we explored subtle ways of structuring the weight matrix without altering the overall distribution of its elements. In particular, we introduced a small random fraction of rows whose elements have smaller-than-average magnitudes. This modification corresponds to a subpopulation of neurons that receive only relatively weak input signals from the rest of the network, and it proved to be a particularly effective stabilization mechanism. As a result of this “minimally invasive,” permutative modification of connectivity, the accuracy becomes excellent even in the largely unbalanced regimes characterized by oscillatory and fixed-point attractors, whereas it shows little improvement in the chaotic regime.

Besides the introduction of “weak rows,” the performance of strongly coupled systems could also be slightly enhanced by introducing other types of patterns into the weight matrix, such as preferentially negative or positive diagonal blocks. By contrast, we also demonstrated that several other possible forms of patterning had no performance-enhancing effect.

Our ultimate goal, however, was to develop a countermeasure against runaway excitations that enables the RNN to perform meaningful information processing regardless of the excitatory/inhibitory balance. This was achieved by augmenting the reservoir computer with an additional unit that implements an Automatic Gain Control (AGC). Although this control unit receives only a global activity signal from the reservoir and sends back only a global modulatory signal into it, the resulting dynamic regulation keeps the RNN in a calm, input-receptive state, irrespective of the balance parameter. Accordingly, the accuracy remains at a very good level across the entire range of balances.

The additional analyses confirm that these conclusions are not an artifact of the original accuracy measure or sequence-generation task. Distribution-oriented performance measures show that AGC strongly reduces both realization-dependent variability and the risk of catastrophic failure. On NARMA-10, which requires non-linear temporal processing and memory, AGC retains most of the performance of the weakly coupled reference while remaining substantially more robust than the unregulated strongly coupled reservoir. Ablation experiments further show that 20 percent weak rows constitute a robust compromise within a broader effective range rather than a uniquely tuned optimum, and that AGC operates successfully over broad ranges of its three control parameters.

The principal findings also generalize substantially across network sizes after scaling the recurrent coupling strength as $1/\sqrt{N}$, although extreme imbalance at *N* = 200 reveals limitations of complete size invariance. Static eigenvalue analysis shows that weak-row stabilization cannot be explained by a reduction of the spectral radius, supporting instead a non-linear mechanism based on heterogeneous recurrent drive and neuronal saturation. Conventional matrix-specific spectral-radius normalization provides an exceptionally strong static baseline for the input-driven sequence task, whereas sparse connectivity produces only partial and balance-dependent stabilization. AGC should therefore be viewed not as universally superior to optimally tuned static normalization, but as an adaptive online mechanism that stabilizes strongly coupled reservoirs without requiring eigendecomposition or a matrix-specific scaling factor.

### 4.2 Outlook

In future work, it would be interesting to test structural and dynamical countermeasures against runaway excitations across a broader range of tasks with different demands on information processing in the reservoir. Another open question concerns which kinds of tasks actually require strong coupling in the first place. In our previous publications, we have already identified several tasks that require only mildly non-linear dynamics and would therefore also work in weakly coupled RNNs. On the other hand, there are tasks such as the prediction of binary systems that may work best in strongly coupled RNNs, where neurons operate in the saturated part of their activation functions and behave more like binary logic gates.

This paper has focused on situations in which runaway excitations arise because the statistical control parameters of the weight matrix—such as the density *d*, the coupling strength *w*, and the balance *b*—are not optimally chosen. A practically even more relevant scenario would involve an RNN whose control parameters are optimized for processing typical inputs, but which can occasionally be pushed into a computationally unfavorable attractor by rare “bad” inputs. For example, the RNN might be tuned close to one of the edges of chaos and normally remain in a healthy dynamical regime, but transition into chaos when exposed to occasional inputs with very large amplitudes. In such cases, it would be particularly interesting to examine whether the AGC mechanism can restore the dynamics to the desired regime within a sufficiently short time interval.

### 4.3 Discussion

The results of the present study connect to several lines of research in reservoir computing, non-linear dynamical systems, and computational neuroscience. In particular, they highlight how structural heterogeneity and dynamical regulation can influence the operating regime of recurrent neural networks and thereby affect their ability to perform input-related information processing.

A central concept in reservoir computing is the so-called echo state property, which requires that the internal state of the network should eventually become determined primarily by the recent input history rather than by arbitrary initial conditions (Jaeger, 2001). In practice, this property is often ensured by constraining the spectral radius of the recurrent weight matrix or by otherwise limiting the effective coupling strength within the reservoir. Subsequent work has further clarified the mathematical conditions under which this property holds and how it relates to the stability of the underlying dynamical system (Manjunath and Jaeger, 2013; Yildiz et al., 2012). Our results suggest an alternative perspective: instead of enforcing a fixed structural constraint on the network parameters, it may be possible to achieve a similar stabilizing effect through dynamical regulation. The automatic gain control mechanism implemented here continuously adjusts the effective coupling strength of the network in response to its momentary activity level. In this way, the system is actively maintained in a dynamical regime that remains sensitive to external input, even when the underlying connection statistics would normally push the network into chaotic or saturating attractor states.

The spectral radius remains an important reservoir-design diagnostic, but it is incomplete for the present non-linear, input-driven system. Weak-row structuring is a permutation of a fixed weight multiset, and the paired eigenvalue analysis in Supplementary Figure S1 shows no systematic reduction of ρ(**W**) that could explain its performance gain. Moreover, **W** describes only the static linear recurrent map. The local dynamics depend on the time-varying Jacobian **J**^(*t*)^ = diag(1−(**y**^(*t*)^)2)*g*^(*t*)^**W**, so tanh saturation, the current state, and the controller gain all matter. AGC consequently has no single static effective matrix or spectral radius. Matrix-specific normalization to a target spectral radius is a very strong baseline for Sequence Generation (Supplementary Figure S4), but it requires eigendecomposition and a selected target for every matrix. Sparse connectivity also helps, while simultaneously reducing recurrent input variance in our uncompensated control. These results do not show that AGC outperforms optimally tuned static scaling; they identify it as an adaptive alternative when online robustness across changing activity and connectivity statistics is desired.

This idea of regulating network dynamics through feedback mechanisms also resonates with observations from biological neural systems. Neural circuits in the brain are not static networks with fixed operating parameters but are subject to multiple forms of homeostatic regulation that maintain their activity within a functional range. Examples include synaptic scaling and other forms of activity-dependent plasticity that adjust the overall excitability of neural populations (Turrigiano, 2012). Inhibitory plasticity has also been proposed as a mechanism that dynamically stabilizes excitation-inhibition balance in cortical circuits (Vogels et al., 2011). More generally, neuronal circuits exhibit a variety of regulatory processes that compensate for parameter variability and maintain stable functional regimes (Marder and Goaillard, 2006). Although the gain control mechanism explored in this work is intentionally simple and operates through a single global control signal, it illustrates how even coarse-grained feedback can stabilize a large recurrent network and extend the regime in which useful computation is possible. In this sense, the mechanism can be interpreted as a minimal computational analog of biological homeostatic regulation.

The same simplicity distinguishes AGC from learned gating. LSTM-family and hierarchical gated networks use task-trained, unit- or channel-specific gates to route and retain information (Hochreiter and Schmidhuber, 1997; Qin et al., 2023; Beck et al., 2024); our controller observes only one permutation-invariant population statistic and returns one global scalar. It cannot implement selective memory routing, but it can stabilize a fixed reservoir without backpropagation through the recurrent dynamics. For a dense reservoir, the recurrent matrix-vector product costs *O*(*N*^2^) per time step. Computing RMS activity and applying one gain to the recurrent-input vector cost *O*(*N*), while updating the running average and exponential feedback costs *O*(1). Total time therefore remains *O*(*N*^2^), controller memory is *O*(1) beyond the reservoir state, and the controller's relative arithmetic overhead decreases approximately as 1/*N*. For a sparse reservoir with *E* non-zero connections, the baseline cost is *O*(*E*); the *O*(*N*) activity measurement can then be non-negligible, especially at fixed mean degree.

The structural countermeasure investigated in this work—the introduction of a small subset of neurons receiving weaker-than-average inputs from the rest of the reservoir—offers a complementary perspective. In strongly coupled networks, runaway excitation typically arises from collective feedback loops that synchronize the activity of large parts of the network. The presence of neurons with weaker incoming connections effectively creates a subpopulation that remains partially decoupled from these global herd effects. Even when the majority of neurons are driven into oscillatory or fixed-point behavior, this weakly coupled subset can still respond to input-related perturbations and thereby preserve useful information in the reservoir state. The results therefore suggest that small degrees of structural heterogeneity can substantially enhance the robustness of reservoir dynamics.

More generally, this observation relates to a broader theme in the study of complex networks: heterogeneous connectivity patterns can prevent the emergence of overly synchronized global dynamics and thereby promote richer dynamical behavior. In biological neural networks, connectivity is known to exhibit pronounced heterogeneity and modular organization (Sporns and Betzel, 2016; Meunier et al., 2010). Such structural diversity has often been associated with enhanced computational flexibility and the coexistence of multiple dynamical regimes within the same network. Although the present study does not attempt to reproduce detailed biological connectivity patterns, the beneficial effect of weakly coupled neuron subsets illustrates how even simple forms of structural diversity can influence the dynamical landscape of recurrent networks.

This broadening of useful parameter regions is consistent with reports that heterogeneity can extend critical-like regimes (Sánchez-Puig et al., 2023). Our ablation nevertheless shows that the effect is conditional rather than monotonic: intermediate weak-row fractions form a broad plateau in strongly unbalanced reservoirs, excessive fractions become detrimental, and the chaotic balanced regime benefits much less. The result supports heterogeneous recurrent drive as a practical stabilizer, not a universal claim that more heterogeneity is always better.

Another aspect of our findings concerns the frequently discussed hypothesis that optimal information processing occurs near the so-called “edge of chaos.” Early studies in reservoir computing and recurrent neural networks suggested that computational capability can peak near transitions between ordered and chaotic dynamical regimes (Bertschinger and Natschläger, 2004; Legenstein and Maass, 2007; Boedecker et al., 2012). Our results confirm that such transition regions can support useful computation in strongly coupled networks. However, they also show that reliance on these narrow parameter regions may not be necessary if regulatory mechanisms are available that actively stabilize the network dynamics. The automatic gain control mechanism explored here effectively replaces the need for precise parameter tuning by maintaining the system in a favorable operating regime over a wide range of structural parameters. From a practical perspective, this suggests that adaptive regulation may represent an alternative design principle for reservoir computers that reduces the sensitivity of the system to the precise choice of network parameters.

This interpretation should be understood in relation to fading memory and the memory–non-linearity trade-off (Dubinin and Effenberger, 2024; Inubushi and Yoshimura, 2017). Weak recurrent coupling promotes reproducible input tracking and forgetting of obsolete states, but may provide insufficient temporal integration or non-linear transformation for demanding tasks. Strong coupling can increase memory and non-linear mixing, yet ultimately produces autonomous chaos or saturation that destroys input sensitivity. AGC shifts the operating point toward the calm side of this trade-off. Its competitive NARMA-10 performance indicates that useful non-linear temporal processing remains, but does not imply that the same controller setting will be optimal for every task.

Finally, the present results also raise several questions for future research. One important direction concerns the interaction between stabilization mechanisms and the specific computational tasks performed by the reservoir. As discussed above, some tasks may benefit from strongly non-linear dynamics or even from saturated neuron states that resemble binary logic elements. Other tasks, particularly those requiring faithful representation of input signals over time, may perform best in calmer, quasi-linear regimes. Understanding how different forms of structural heterogeneity or dynamical regulation influence the trade-off between stability and non-linear computational power remains an open problem. In this context it may also be informative to relate the present observations to studies showing that trained recurrent networks often operate on low-dimensional dynamical manifolds embedded in the high-dimensional state space (Sussillo and Barak, 2013).

The scope of the present validation is deliberately limited. We did not calculate formal linear memory capacity; NARMA-10 is a functional benchmark combining memory and non-linearity and is not equivalent to such a capacity measurement. We also did not compare intrinsic plasticity, because adapting neuron-specific activation functions raises a distinct mechanistic question that warrants a dedicated study. The Supplemental controls instead isolate two conventional static interventions, spectral-radius scaling and sparse connectivity. Accordingly, the results establish robustness for two complementary tasks and the tested parameter ranges, not universal superiority across reservoir architectures or computational objectives.

The size-control experiment provides an initial, qualified scalability test. Scaling weight width as $1/\sqrt{N}$ approximately preserves recurrent-input variance and the balanced random-matrix eigenvalue bulk, and the principal performance patterns persist for *N* = 50, 100, and 200. This rule does not preserve the coherent mean mode generated by strong E/I imbalance. Consistent with that limitation, AGC remains highly robust around balanced connectivity but performance declines for some strongly positive *N* = 200 cases, including a pronounced realization-dependent tail. Thus, the data support substantial size generalization, not complete size invariance or asymptotic scaling. Much larger reservoirs, sparse implementations, and more complex tasks remain to be tested. Adaptive regulation may be particularly useful in physical and neuromorphic systems subject to parameter variability and environmental fluctuations (Tanaka et al., 2019).

Taken together, the present study demonstrates that runaway excitation in strongly coupled recurrent neural networks can be mitigated either by introducing subtle structural heterogeneity or by implementing a simple form of global dynamical regulation. Both strategies substantially enlarge the dynamical regime in which reservoir computers remain sensitive to input signals and capable of reliable information processing. These findings suggest that combining structural diversity with adaptive regulation may represent a promising direction for the design of robust reservoir computing systems.
