# Supplementary material for: Structural and dynamical strategies to prevent runaway excitation in reservoir computing
Source: Front Comput Neurosci. 2026 Aug 14;20:1845838. doi: 10.3389/fncom.2026.1845838 (PMC13522119; doi:10.3389/fncom.2026.1845838)
Supplement: Supplementary file 1 [file Data_Sheet_1.pdf]

Supplemental Material

Static Eigenvalue Spectra of Homogeneous and Weak-Row Reservoirs

To test whether weak-row structuring stabilizes the reservoir through a simple reduction of the recurrent matrix spectrum, we compared homogeneous and weak-row matrices at fixed network size $N = 50$, coupling width $w = 1$, and connection density $d = 1$. The balance was scanned at 33 values from $b = -1$ to $+1$, with ten paired random realizations per value. For each realization, the weak-row matrix was obtained by rearranging the elements of the corresponding homogeneous matrix, so that the two conditions contained exactly the same set of weights. We calculated all complex eigenvalues and the static spectral radius $\rho(\mathbf{W}) = \max_i |\lambda_i|$.

The spectral radius exhibited a pronounced V-shaped dependence on balance, with its minimum near $b = 0$ and values of approximately 40 at the fully inhibitory and fully excitatory limits. Weak-row structuring changed this curve only marginally. At $b = 0$, for example, the mean spectral radius was 7.39 for homogeneous matrices and 7.47 for weak-row matrices, and the largest difference between the two mean curves over the complete balance scan was only about 0.24. Thus, the large performance improvement produced by weak rows is not accompanied by a systematic reduction of the static spectral radius.

The complex-plane spectra in Fig. S1 support the same conclusion. At $b = 0$, both conditions show an approximately circular eigenvalue bulk around the origin. At $b = -0.5$ and $b = +0.5$, respectively, a large negative or positive real outlier emerges and dominates the spectral radius, while the central bulk remains qualitatively similar. Neither the bulk nor the real outlier changes substantially after the weak-row rearrangement. This negative result is consistent with the nonlinear, state-dependent nature of the reservoir dynamics: the relevant local Jacobian contains both $\mathbf{W}$ and the instantaneous derivatives of the tanh activation functions. Weak rows therefore appear to stabilize computation by redistributing recurrent drive and preserving a responsive neuronal subpopulation, rather than by globally shrinking the static spectrum.

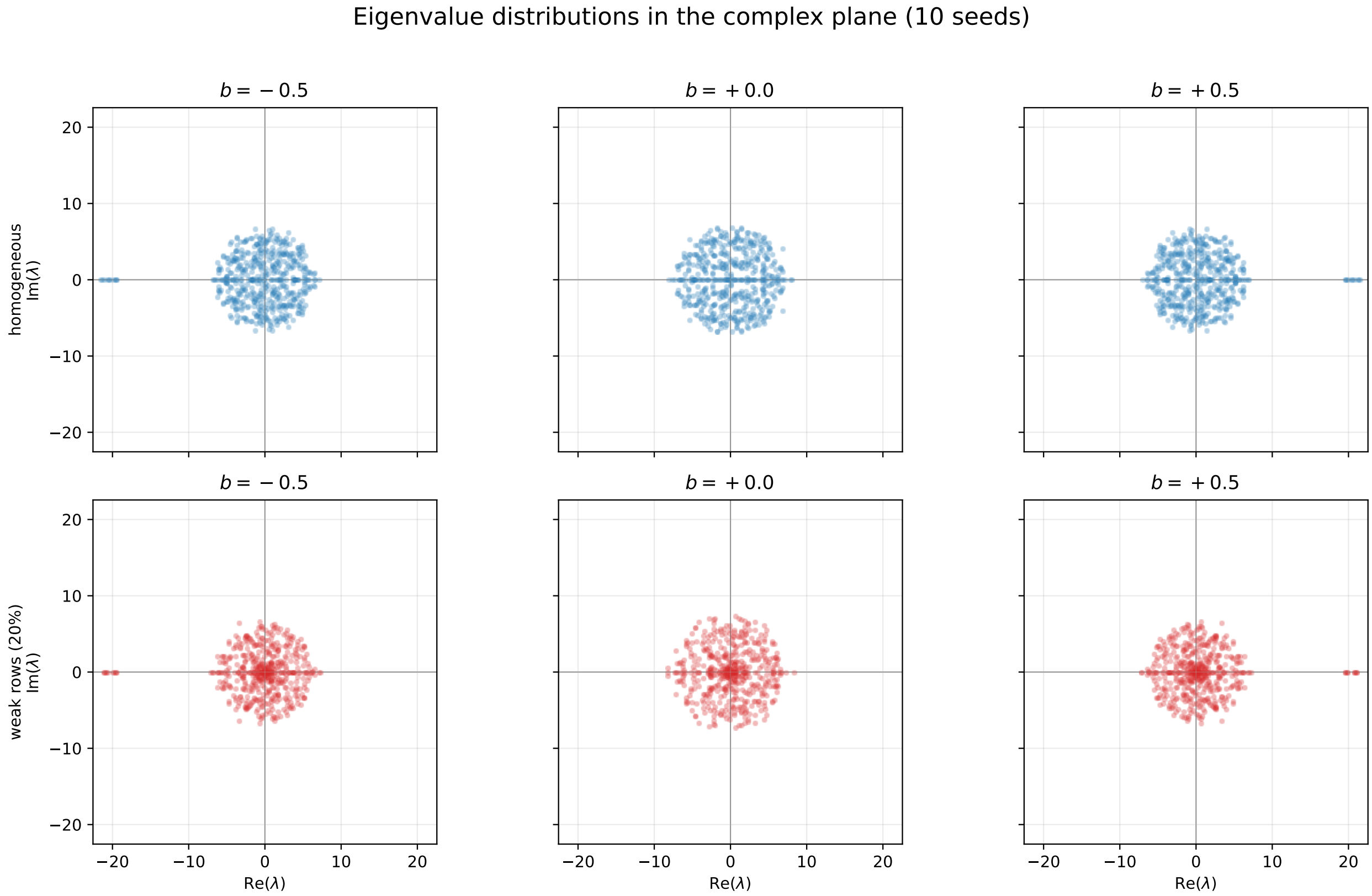

Figure S1: Complex-plane eigenvalue distributions for balances $b = -0.5$, 0, and $+0.5$, comparing homogeneous reservoirs with reservoirs containing weak rows.

NARMA-10 Performance Across E/I Balance

We used NARMA-10 to test whether the comparatively calm operating regime produced by AGC retains sufficient nonlinearity and memory for a standard nonlinear temporal benchmark. The calculation comprised 17 balance values from $b = -1$ to $+1$ and 50 paired random seeds per balance. Each run used independent training and test sequences of 5,000 time steps, with the first 200 steps excluded as washout. For every seed, the calm, homogeneous, weak-row, and AGC conditions re-ceived identical NARMA inputs; the latter three conditions additionally originated from the same strongly coupled recurrent matrix.

Figure S2 shows individual-run and mean accuracy curves, where $A = 1/(1 + \text{NRMSE})$ and $A = 0.5$ corresponds approximately to the constant target-mean baseline. At balanced connectivity, the mean accuracy was 0.698 for the calm reservoir and 0.673 for AGC, whereas the homogeneous and weak-row conditions remained near baseline at 0.498 and 0.500, respectively. The best mean values were 0.704 for the calm reservoir at $b = +0.125$ and 0.681 for AGC at the same balance. Thus, AGC retains most of the NARMA-10 performance of the weakly coupled reference despite acting on a strongly coupled recurrent matrix.

Across all balance-seed combinations, the calm and AGC reservoirs exceeded $A = 0.5$ in 99.3 percent of runs. The corresponding fractions were 38.0 percent for the homogeneous reservoir and 88.9 percent for weak rows. AGC also produced only one catastrophic outcome with $A < 0.1$, compared with 15 for the homogeneous, four for the weak-row, and five for the calm condition. Some mean curves remain irregular even with 50 seeds because rare severe failures create a heavy lower performance tail; for example, at $b = +0.25$ the weak-row condition has mean accuracy 0.481 but median accuracy 0.551. The robust distributional measures in Fig. 5(b) account explicitly for this asymmetry. Overall, the NARMA-10 results do not support the concern that AGC removes task-relevant nonlinear temporal processing.

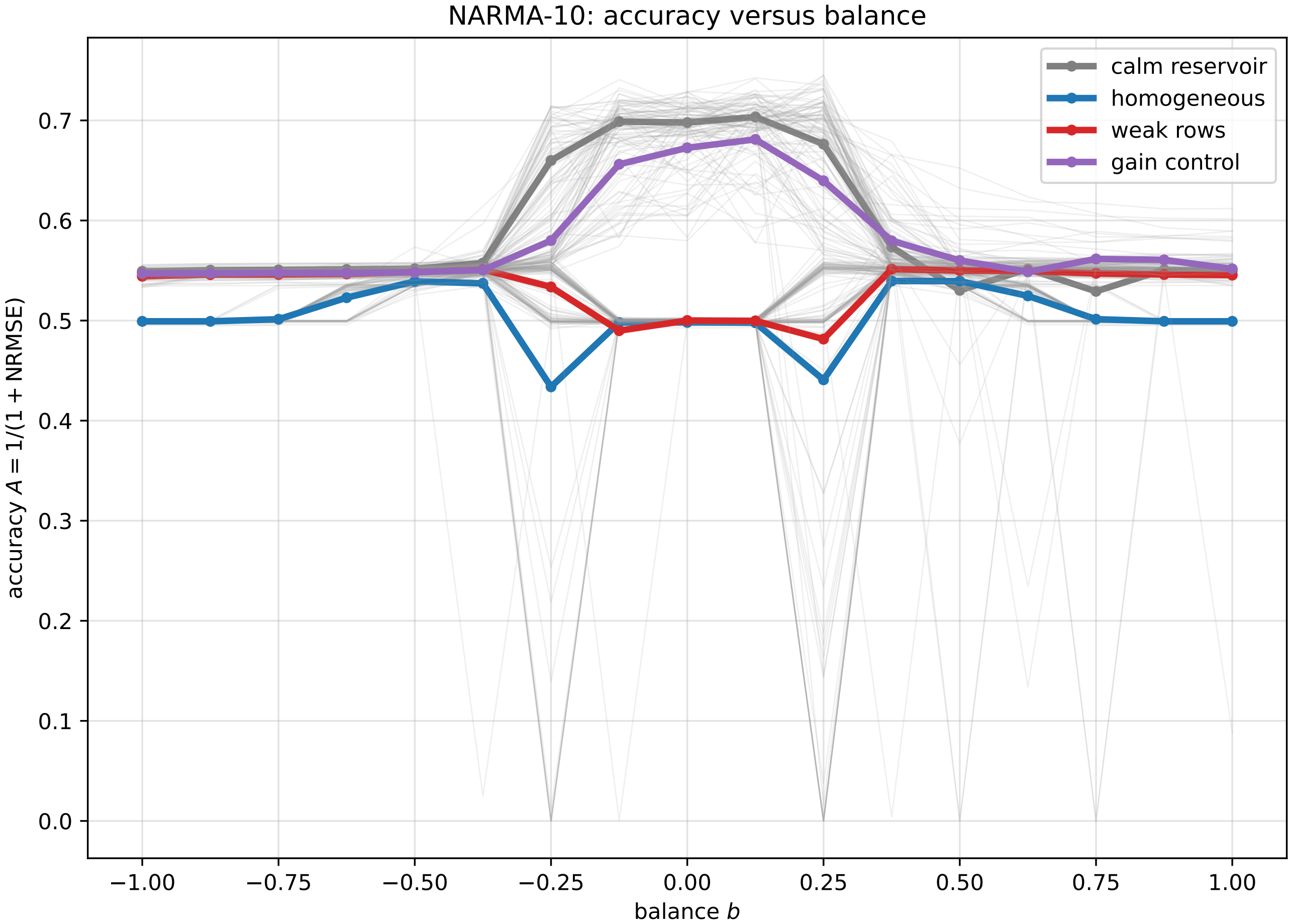

Figure S2: NARMA-10 accuracy as a function of excitatory/inhibitory balance for the four reservoir conditions. Thin curves show individual realizations and thick colored curves show averages over 50 random seeds.

Network-Size Generalization

We repeated the sequence-generation experiment for N = 50, 100, and 200 to assess whether the principal findings generalize beyond the original network size. To approximately preserve the variance of recurrent input and the random-matrix eigenvalue bulk, the recurrent-weight width was scaled as $w(N) = w(50)\sqrt{50/N}$. The strongly coupled conditions therefore used w = 1, 0.707, and 0.5, while the calm references used w = 0.1, 0.0707, and 0.05. For each size, 17 balance values and 20 paired seeds were evaluated, with the weak-row fraction and AGC parameters kept fixed. Figure S3 shows median log10(NRMSE) and interquartile ranges for all four reservoir conditions.

Near balanced connectivity, the results are remarkably consistent across size. The calm reservoir remains nearly perfect, with median accuracies of 0.9998, 0.9999, and 1.0000 for N = 50, 100, and 200 at b = 0. The homogeneous strongly coupled reservoir remains close to the elementary baseline at the same balance, with median accuracies between 0.514 and 0.518. Weak rows provide a modest central improvement and can produce nearly perfect performance in substantial unbalanced regions at every size, although the locations and widths of these regions shift with N. In particular, the N =200 weak-row curve contains sharp transitions and an isolated severe failure near b = +0.125.

AGC is the most size-robust of the strongly coupled conditions around balanced connectivity: at b = 0, median accuracy is 0.962, 0.965, and 0.961 for the three sizes. For N = 50 and N = 100, it remains consistently high across the full balance range. At N = 200, performance is still excellent for negative and near-balanced connectivity but declines for strongly positive balance, reaching approximately 0.59–0.69 from b = +0.5 to +1. This qualified generalization reflects a limitation of the $1/\sqrt{N}$ scaling rule: it controls the random eigenvalue bulk but not the nonzero-mean coherent mode induced by E/I imbalance, whose magnitude can grow approximately as $\sqrt{N}$. The results therefore support substantial size generalization without implying complete size invariance under extreme imbalance and fixed controller parameters.

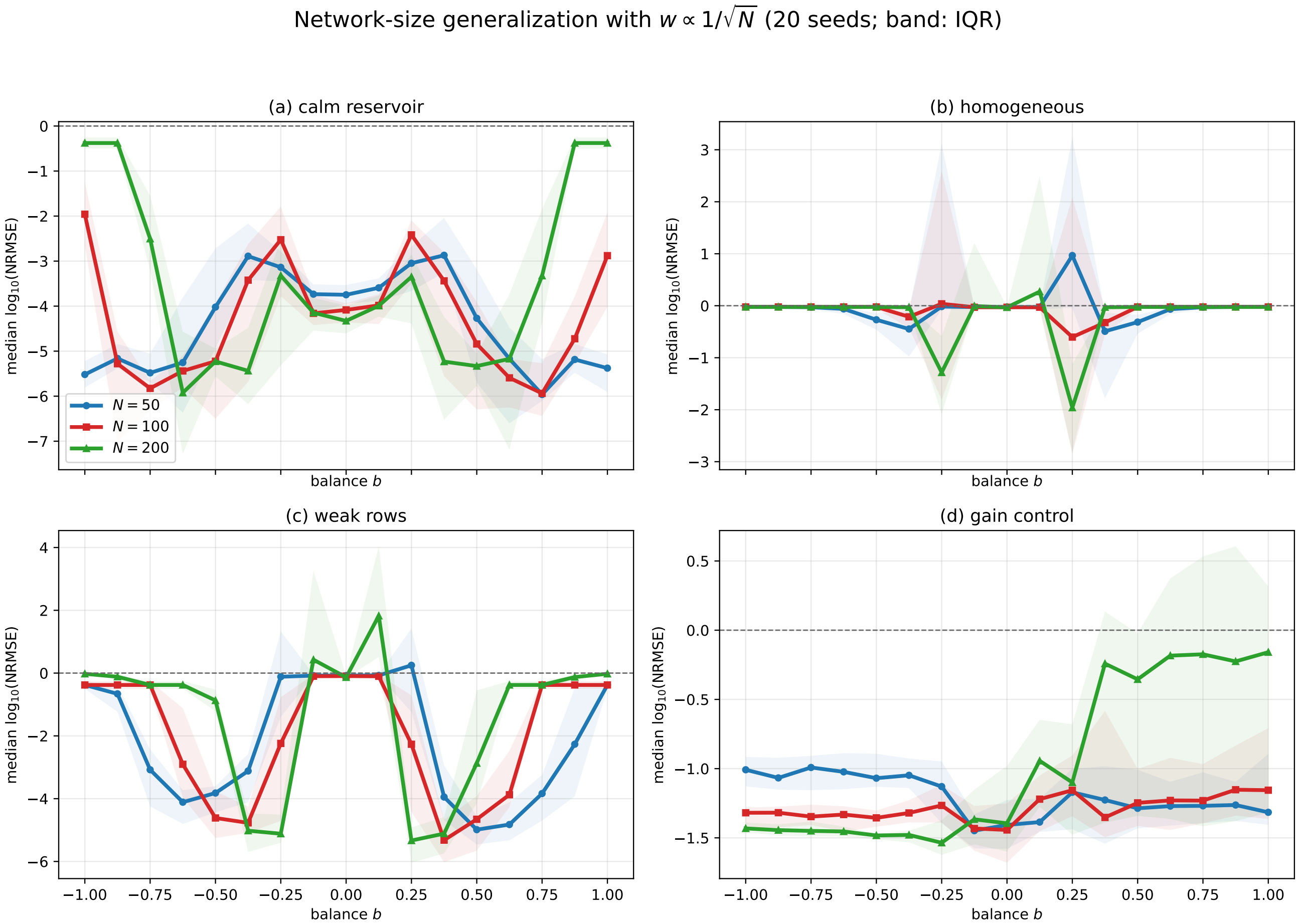

Figure S3: Sequence-generation performance for reservoir sizes $N = 50$, 100, and 200 after scaling recurrent coupling widths as $1/\sqrt{N}$. Curves show median $\log_{10}$(NRMSE) and shaded regions show interquartile ranges over 20 random seeds.

Static Stabilization by Spectral-Radius Scaling and Sparse Connectivity

As static stabilization baselines, we compared matrix-specific spectral-radius scaling with sparse recurrent connectivity on the sequence-generation task. The scan used $N = 50$, an unscaled recurrent-weight width $w = 1$, 17 balance values, and 50 paired seeds per balance. In the first baseline, each dense matrix was multiplied by a global factor such that its spectral radius became $\rho_{\text{target}} = 0.5$, 0.7, 0.9, or 1.1. In the second baseline, recurrent densities $d = 0.1$, 0.2, 0.5, and 1 were generated with nested masks. The retained weights were not variance-compensated, so decreasing density reduces both the number of connections and the total recurrent input variance.

Scaling each matrix to a prescribed spectral radius almost completely removes the balance dependence of performance (Fig. S4(a)). Every tested target yields median accuracy above 0.969 at every investigated balance. At $b = 0$, median $\log_{10}(\text{NRMSE})$ is -5.344, -4.076, -2.637, and -1.504 for target radii 0.5, 0.7, 0.9, and 1.1, respectively. The systematic advantage of the smaller radii is much clearer in the logarithmic error representation than in accuracy, where all curves are com-pressed near one. For this strongly input-driven task, matrix-specific global weakening is therefore an exceptionally effective static solution.

Sparse connectivity also improves performance, but less uniformly (Fig. S4(b)). At $b = 0$, median accuracy is 0.697, 0.544, 0.514, and 0.511 for densities 0.1, 0.2, 0.5, and 1, respectively. The lowest density gives the most robust improvement in the central balance region and has a worst median accuracy of 0.671 over the scan. Intermediate and dense networks, by contrast, retain narrow peaks, abrupt balance-dependent transitions, and broad interquartile ranges. Sparsity is consequently a useful but parameter-sensitive static intervention.

These baselines stabilize the reservoir in ways that differ fundamentally from AGC. Spectral-radius scaling uses complete knowledge of each realized matrix to impose a preselected global eigenvalue bound, whereas sparsity acts indirectly by removing connections. AGC instead changes effective recurrent coupling online in response to the observed network activity and requires neither eigendecomposition nor a matrix-specific prefactor. The present result should therefore not be interpreted as AGC outperforming an optimally scaled static reservoir. Rather, it shows that static matrix normalization can solve this particular input-driven task extremely well, while AGC provides an adaptive mechanism intended for situations in which the effective operating regime can change over time.

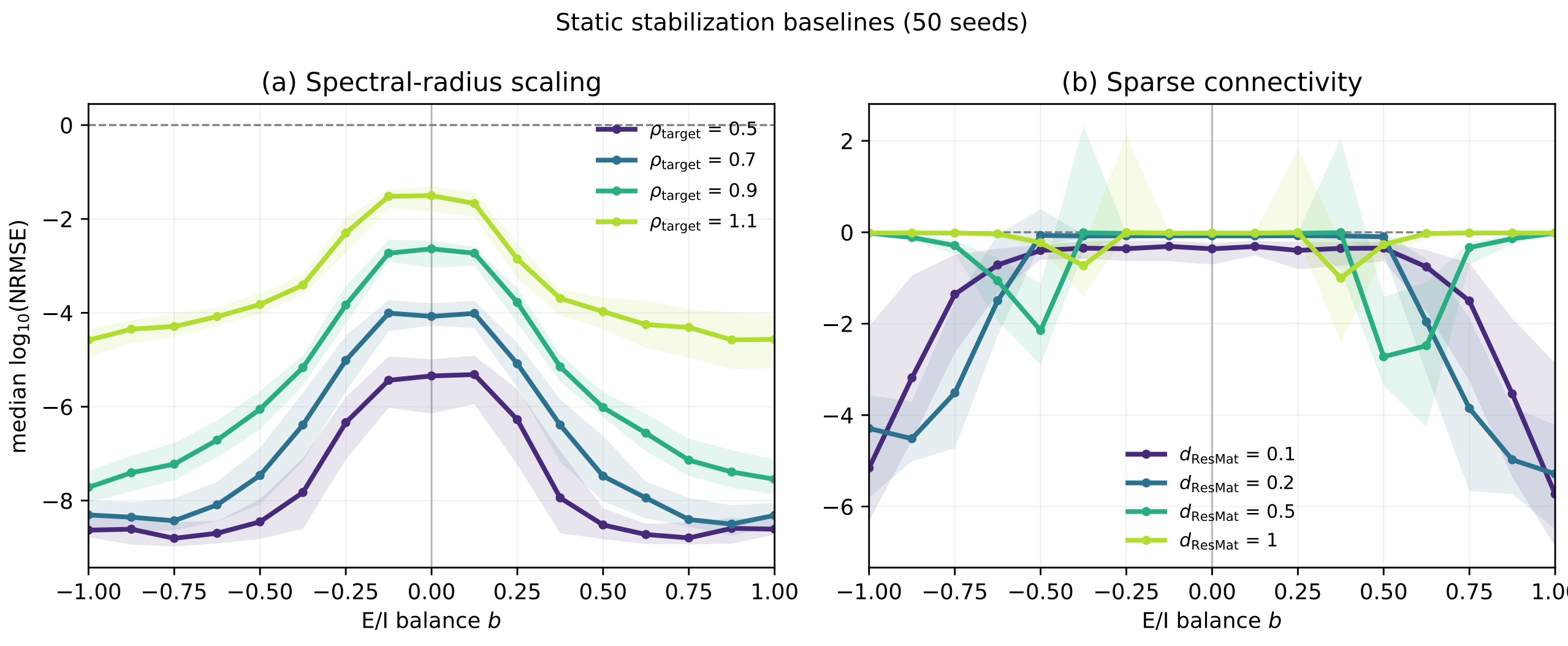

Figure S4: Sequence-generation performance under static stabilization. Panel (a) compares four target spectral radii, and panel (b) compares four recurrent connection densities. Curves show median $\log_{10}(\text{NRMSE})$ and shaded regions show interquartile ranges over 50 random seeds.
